# Efficient visualization of chemical space

**DOI:** 10.1039/d6sc03716j

**Published:** 2026-09-11

**Authors:** Akash Surendran, Krisztina Zsigmond, Lexin Chen, Ramón Alain Miranda-Quintana

**Affiliations:** a Department of Chemistry, Quantum Theory Project, University of Florida Gainesville FL 32611 USA quintana@chem.ufl.edu ak.surendran@ufl.edu

## Abstract

The concept of chemical space is critical in cheminformatics, medicinal chemistry, and machine learning applications. Despite this, the high dimensionality of molecular representations greatly complicates its sampling, analysis, and visualization. A popular approach to overcome problem is to project these representations to a “human-manageable” subspace, usually containing only two dimensions. Non-linear dimensionality reduction techniques are by far the preferred strategy, following the reasoning that their flexibility can accommodate any arbitrary distribution originally present in the high-dimensional space. However, this ignores the elevated computational cost of these methods and the difficulty in tuning their hyper-parameters. Here, we show that basic properties of the metrics used in the original space can be used to infer the shape of the chemical space, which in turns suggests an optimal strategy to project chemical information to lower dimensions. The key insight is to realize that no matter the set of molecules, the binary fingerprint representation evaluated under the Tanimoto metric can be considered to lie on a hyper-spherical surface. The smooth nature of this manifold indicates that we can use clustering in the original fingerprint representation to identify locally-dense sectors of chemical space, and selectively project them simply using linear (hyper-parameter free) methods, like principal component analysis. This approach surpasses non-linear techniques in several neighborhood preservation metrics, while only requiring a fraction of the computational cost. This pipeline is implemented in our N-Ary Mapping Interface (NAMI: https://github.com/mqcomplab/NAMI), which we tested in the visualization of tens of millions of molecules.

## Abstract 2

The concept of chemical space is critical in cheminformatics, medicinal chemistry, and machine learning applications.

## Graphical abstract

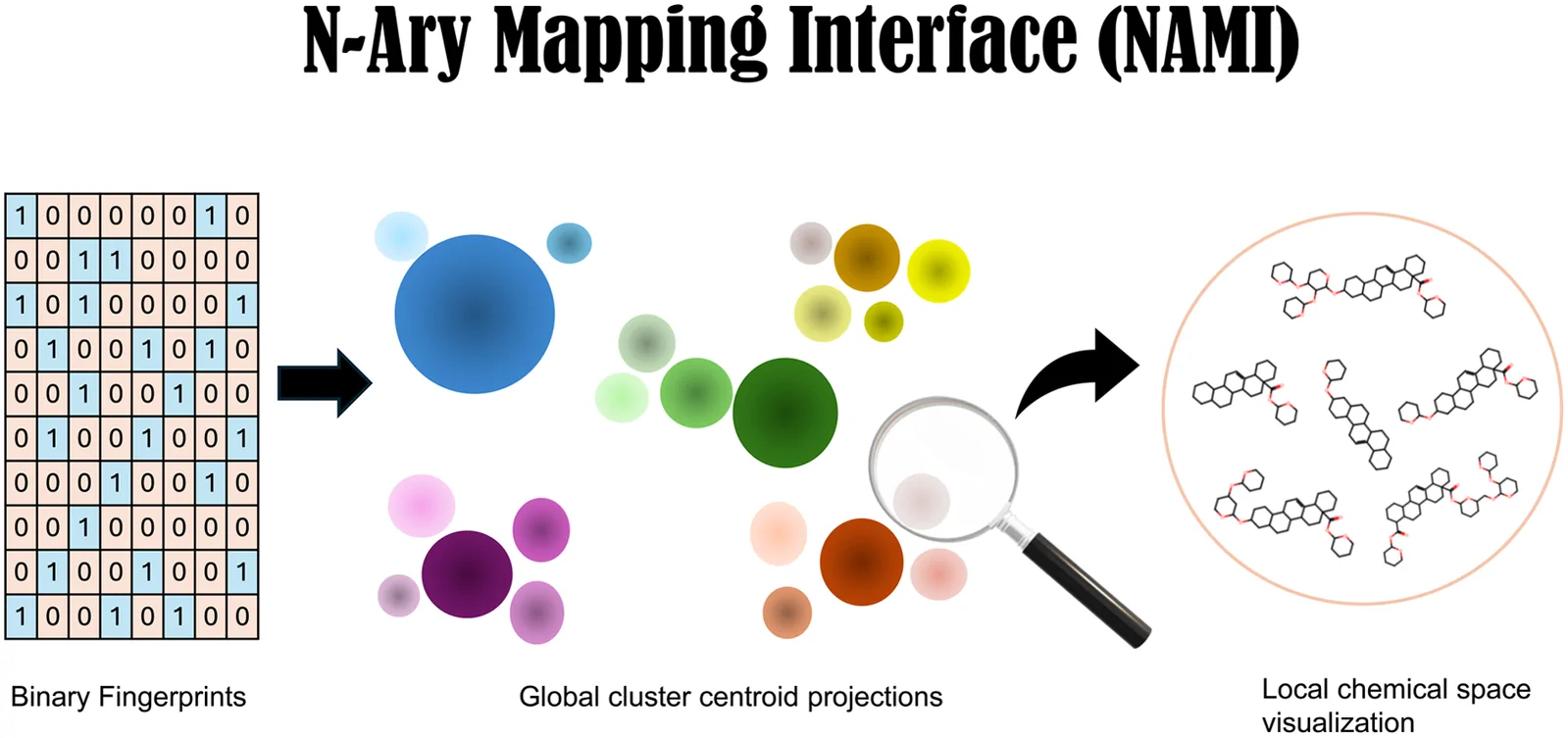

## 1 Introduction

It is a truth universally acknowledged, that a chemist in possession of a large dataset, must be in want of a non-linear projection method to analyze it.^1–7^ This is a consequence of the high dimensionality of the vector representations commonly employed to encode molecular information,^8–10^ ranging from thousands of bits in popular fingerprint formats to larger arrays of molecular descriptors, which cannot be directly interpreted, since the similarity tools^11–14^ used to work with bit-vectors are far removed from our intuition about the Euclidean metric spaces. This poses a key problem: how to represent the chemical space in a way that is easy to navigate and interpret? The ever-increasing size^15–20^ of synthetically and theoretically accessible chemical space complicates this task, since the computational cost of representation methods can quickly become prohibitive. Beyond visualization, the effectiveness of ML models is intrinsically linked to how these representations capture structure and property-based information.^21–24^ Molecules close together in chemical space share similar properties and biological activities critical for ligand-based virtual screening and drug discovery^25–34^ and hence, mapping chemical space can narrow down billions of possibilities into a manageable set for experimental verification.

Despite the importance of chemical space in modern drug design and ML studies, it is worth noting that in the vast majority of cases, this notion refers to a “collection” of molecules, rather than an actual mathematical “space”.^35–39^ Virtually no pre-existing structure is considered when dealing with chemical libraries, which complicates their analysis. When visually inspecting a compound dataset using dimensionality reduction (DR) methods, they usually ignore any pre-existing characteristics in the high-dimensional space. For similarity-based visualization purposes, DR quality is evaluated through conservation of neighborhood information from high to low-dimensional representations, which is why PCA^40–44^ is usually discouraged in favor of t-SNE,^45–50^ UMAP,^44,51–54^ and GTM,^55–58^ since the former performs only a linear projection while the latter make no assumptions about the high-dimensional manifold. This flexibility, however, comes at a steep price; these non-linear methods like t-SNE and UMAP carry substantial computational cost and assume Euclidean relations between the inputs, a non-trivial assumption for binary fingerprints. These limitations have not dampened their popularity, as t-SNE and UMAP have been applied to drug-like molecular similarity visualization,^59^ reaction mapping,^48^ and systematic benchmarks of DR quality in chemical applications.^60^ A central challenge across all these studies is the hyperparameter sensitivity: optimal settings are highly dataset-specific, depending on molecular diversity, fingerprint type, and sample size, and given the computational costs, exhaustive tuning is often infeasible for large datasets. As t-SNE seeks to minimize the KL divergence between pairwise similarities in high and low-dimensional space, its complexity scales quadratically with dataset size. Recently, we demonstrated that sampling ∼10% of a large dataset is sufficient to obtain optimal hyperparameters for non-linear DR methods,^61^ yet the fundamental non-universality of these tools motivates the need for alternatives requiring no hyperparameter tuning.

In this paper, we explore two related issues: how simple properties of the metric used in high-dimensional space can improve low-dimensional projections and how clustering can be used to make linear DR methods more faithful and interpretable than t-SNE, UMAP and GTM. DR and clustering are routinely paired, with DR commonly used as a pre-processing step before clustering to reduce computational cost and aid visual tuning of partition hyperparameters. However, this practice can mask deeper issues: performing DR before clustering fails to capture the true inter-molecular relations in the original space, while biasing users toward easy-to-spot patterns in low dimensions. Here, we show that simply reversing this recipe (performing the clustering in the original space and then doing the DR) considerably improves the quality of the final projections, to the point where PCA even surpasses non-linear alternatives. This not just allows to obtain better low-dimensional chemical representations, but it does so at a fraction of the computational cost of current approaches, to the point that multi-million libraries can now be fully processes in minutes in a single workstation. To conclude, we provide a package (N-Ary Mapping Interface, NAMI) that implements this pipeline, which is freely available at: https://github.com/mqcomplab/NAMI.

## 2 Methods & systems

### 2.1 Local neighborhood metrics

As noted by Orlov *et al.*,^60^ DR methods need to be carefully and quantitatively evaluated on how well they preserve high-dimensional neighborhood information. Here we employ several complementary metrics to assess this. The Average Nearest Neighbor Preservation (*P*_NN_) checks the consistency of the *k* nearest neighbors of each point across both spaces. The Co-*k* Nearest Neighbor Size (*Q*_NN_) tracks shift in rankings of the first *k* neighbors, with the area under this curve (AUC) summarizing global neighborhood preservation across all possible neighborhoods. A more detailed picture is provided by the local continuity meta criterion, which is a normalized version of *Q*_NN_, favoring local information by penalizing distant neighbor calculations. Additionally, *Q*_local_ and *Q*_global_ (respectively, the integral of the LCMC from 0 to a given *k*_max_, and from *k*_max_ to infinity) dissect the information contained in LCMC into local and global components. Trustworthiness quantifies the false neighbors introduced in low dimensions while continuity captures the complementary error. All these metrics were evaluated using the benchmark code developed by the Varnek group, which is freely available at https://github.com/AxelRolov/cdr_bench.

### 2.2 Clustering large chemical spaces

The clustering algorithm to be used prior DR should satisfy three key criteria: (1) no assumed pre-distribution of molecules, (2) robust identification of tight, high-similarity subsets, (3) computational efficiency for large chemical spaces. For these reasons, we employ BitBIRCH,^62,63^ which requires a single user-defined parameter (similarity threshold) and is it is guaranteed to find clusters at least as compact as the given threshold. BitBIRCH scales linearly with the number of molecules, and its defining characteristic is the reduction of each cluster's information to a condensed Bit Feature (BF) containing the number of molecules *N*_*j*_, their indices mols_*j*_, and column wise sum of cluster fingerprints **ls**_*j*_ (with centroid **c**_*j*_ stored as well for convenience), making it suitable ultra-large libraries. Cluster membership is determined here using the diameter criterion: a new molecule is added to a cluster if the average pairwise Tanimoto similarity exceeds the threshold. This average can be computed directly from the BF *via* the iSIM framework:^64–67^1
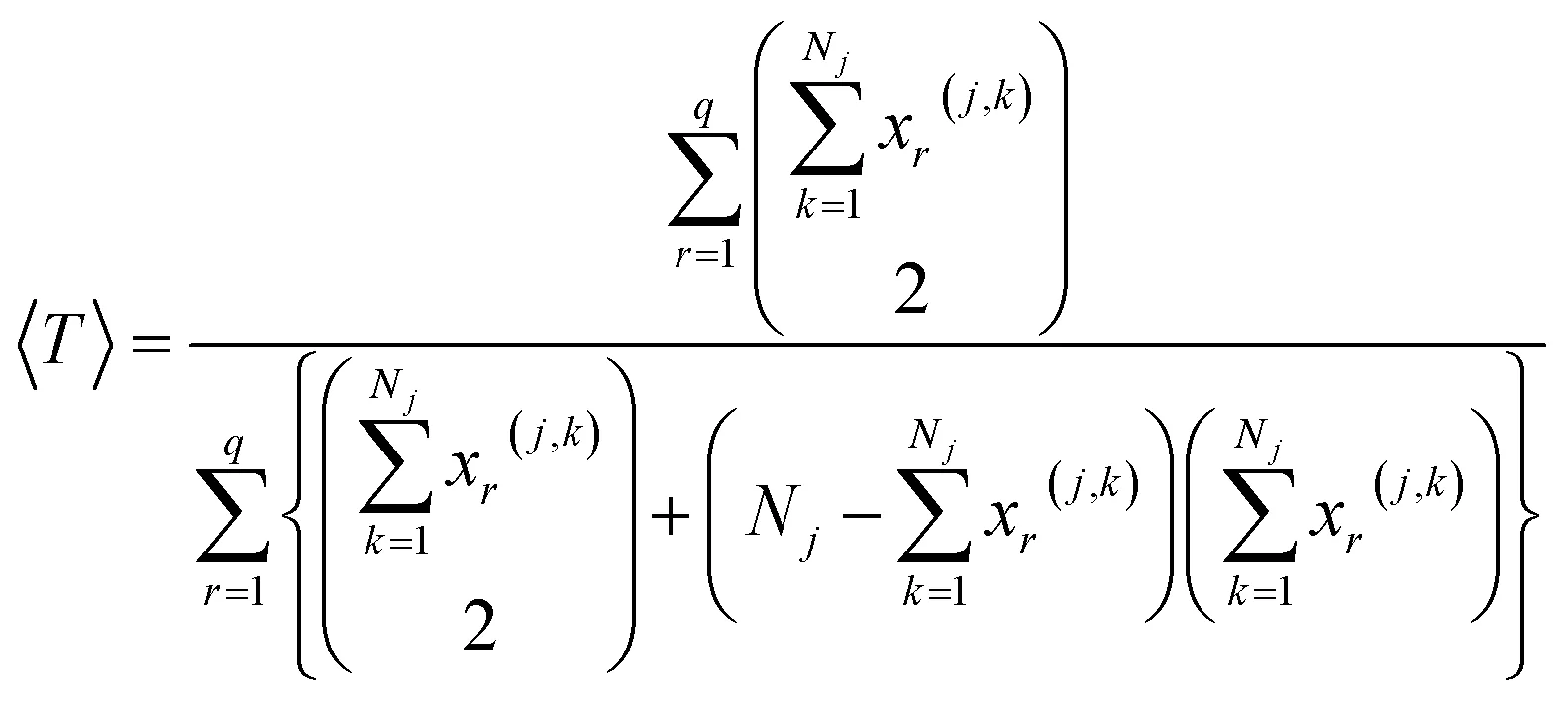
where 
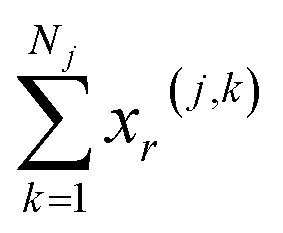
 is the *r*^th^ component of **ls**_*j*_.

Recently, we introduced BitBIRCH-Lean (bblean), a faster and memory-efficient implementation of the original BitBIRCH clustering algorithm built on four primary optimizations. First, dynamic overflow checking enables the usage of compact uint8 arrays instead of int64, combined with native Numpy bitwise operations to bit-pack fingerprints, reducing memory per-molecule by 8-fold. Third, computationally intensive Tanimoto calculations and node splitting operations are performed as C++ extensions *via* pybind11. Finally, bblean overcomes the sequential processing bottleneck by clustering fingerprints in parallel batches in partial trees which are refined and merged into a single unified tree. Collectively, this yield a 30-fold reduction in RAM usage, allowing a mid-range workstation to cluster 1 billion molecules in just 2.5 hours using just 55 GB of RAM, a task that previously took over 2 TB of memory and multiple days.

### 2.3 Cartography

As a simple model to study the projections of non-linear high-dimensional manifolds into planar surfaces (with particular relevance to fingerprint representations, as seen in Section 3.1), we will consider the process of mapping the surface of the unit sphere in 
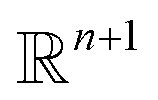
 (centered at the origin of coordinates), defined as: 
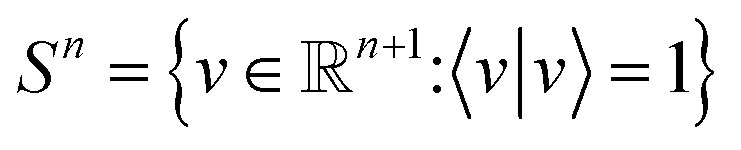
. In particular, we will focus on the gnomonic and stereographic projection (Fig. 1).

**Fig. 1:**
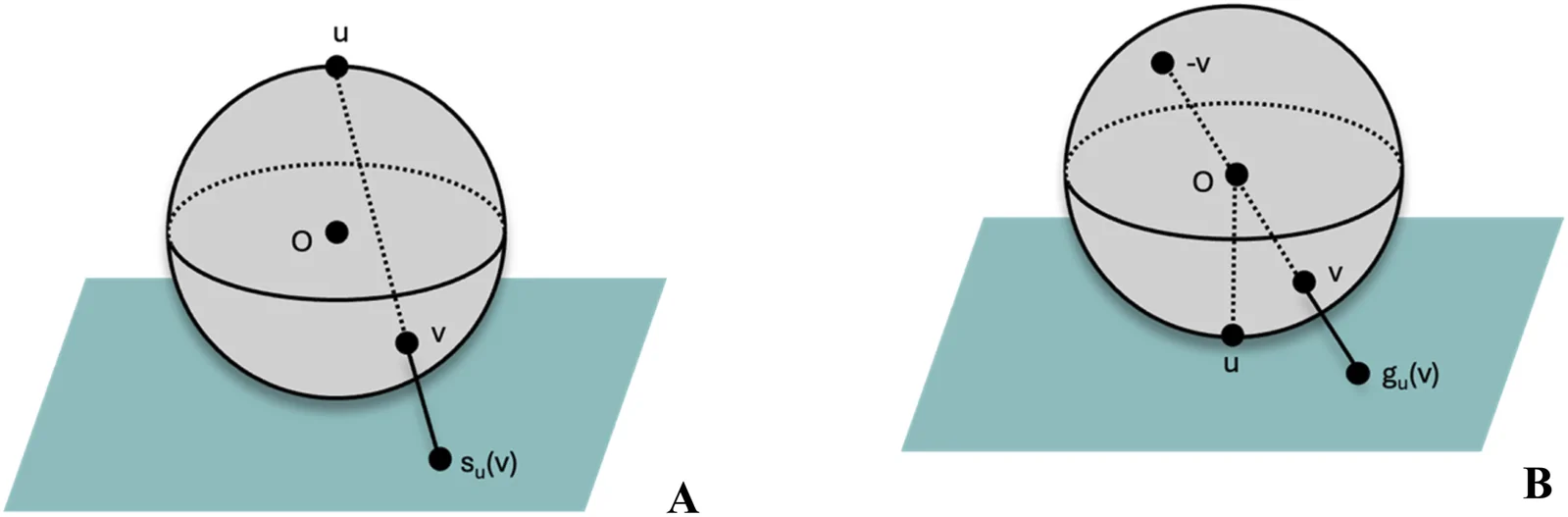
Cartoon representation of the stereographic (A) and gnomonic (B) projections.

The gnomonic projection is built starting from a vector *u* in *S*^*n*^as:^68^2
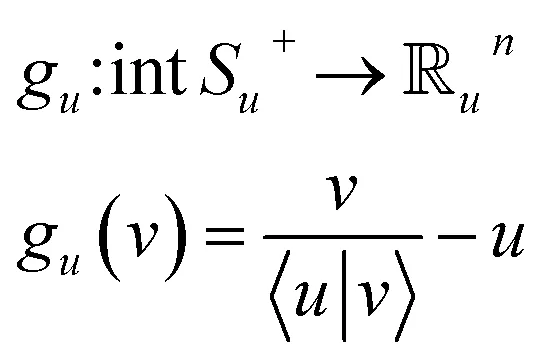
where *S*_*u*_^+^ = {*v* ∈ *S*^*n*^:〈*u*|*v*〉 ≥ 0}.

Notice that although the projection is defined for vectors orthogonal to *u*, the projection of an orthogonal vector *v*, will have infinite components (this happened for some of the molecules in the ChEMBL natural products set, when represented with ECFP4 fingerprints, see below).

The stereographic projection^69^ is often defined with respect to one of the unit vectors (*e.g.*, *e*_1_ = (1, 0, …, 0)). In that case, the projection hyperplane 
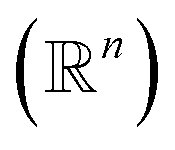
 is:3
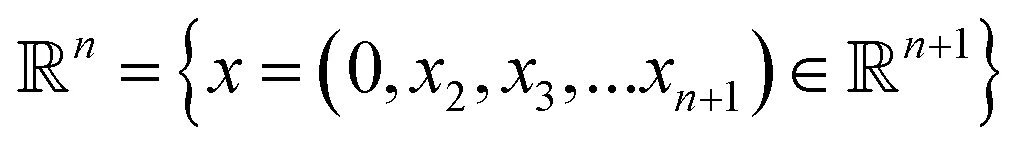


Resulting in the projection:4
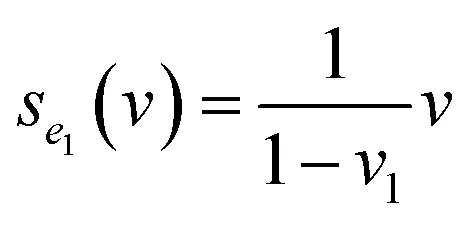
where *v*_1_ is the component of *v* in *e*_1_. The stereographic projection then connects the points *u*, *v*, and *s*_*u*_(*v*) with a line (see Fig. 1A). Namely:5
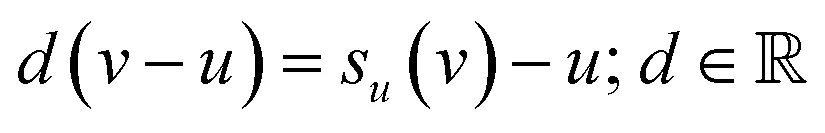
with:6
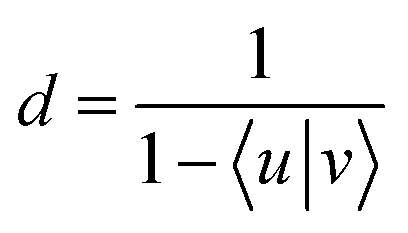
Here, we consider two possibilities for the reference vector *u*: the normalized (1, 1, 1, …) vector or the medoid of the set. Similarly, we use (−1, −1, …) and -medoid as references for the stereographic projection, since the projection plane in this case is on the opposite side of the unit sphere. Note that, to avoid over-complicating the ensuing discussions, we will still refer to these projections as stereographic 111 and stereographic medoid, instead of stereographic −1, −1, −1 and stereographic -medoid.

### 2.4 Datasets and DR methods

For the comparison of neighborhood preservation metrics across non-linear methods (t-SNE, UMAP and GTM) and PCA, a dataset CHEMBL219_Ki curated by Grisoni *et al.*^70^ on Dopamine D4 receptor containing 1859 molecules as SMILES strings was used. We used a small dataset to ensure that the comparisons could be completed within a suitable computational time, since non-linear methods like t-SNE and GTM do not scale linearly with number of molecules and hence could be infeasible for larger datasets.

The projections and local neighborhood analysis were performed on the CHEMBL33 natural products database with 64 086 molecules.^71,72^ Two types of binary fingerprint representation were employed: Extended-Connectivity Fingerprints (ECFP)^73^ with radius 2 and 1024 bit length, and the 2048 bit RDKit fingerprints. Finally, to visualize a large dataset we use a random subset of ZINC20 (ref. 74) containing 10 million compounds, prepared primarily for deep docking applications and provides clustered protonation ready SMILES strings. CHEMBL33 and CHEMBL219_Ki are available as csv files on the GitHub for this paper https://github.com/mqcomplab/NAMI and the ZINC20 dataset can be downloaded from the website: https://files.docking.org/zinc20-ML/smiles/ as ZINC_20_smiles_chunk2.

The hyperparameter grid for t-SNE, UMAP and GTM was chosen to be the same as Orlov *et al.*^60^ The t-SNE: perplexity: [1, 2, 4, 8, 16, 32, 64, 128] and exaggeration: [1, 2, 3, 4, 5, 6, 8, 16, 32], for UMAP: nearest neighbors: [2, 4, 6, 8, 16, 32, 64, 128, 256] and minimal distance: [0.0, 0.1, 0.2, 0.3, 0.4, 0.6, 0.8, 0.99] and for GTM: number of nodes: [225, 625, 1600], number of basis functions: [100, 400, 1225], regularization coefficients: [1, 10, 100] and basis widths: [0.1, 0.4, 0.8, 1.2]. The hyperparameters were tuned to preserve the maximum percentage of nearest 20 neighbors.

## 3 Results

### 3.1 Tanimoto similarity and Euclidean distance

As noted above, one of the often-ignored problems plaguing DR studies is that even though the original fingerprints are compared using the Tanimoto index,^75^ the standard DR process itself starts by calculating the separation between compounds using the Euclidean distance. However, given that most data-processing algorithms (including DR methods) are optimized to operate over vectors with continuous entries, it is convenient to have a transformation that maps binary fingerprints to real vectors, while trying to preserve the ranking of similarities. To achieve this, we first have to consider the Tanimoto formula between two molecules:7
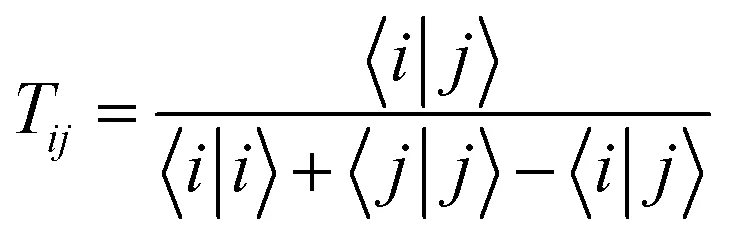


From here, it is clear that all molecules in “fingerprint space” are equidistant from a fingerprint consisting of only zeroes (∀*i*; 〈0|*i*〉 = 0). This provides a key revelation into the shape of chemical space, since the use of the Tanimoto similarity directly implies that the molecules lie in the surface of a multi-dimensional sphere. We can use this insight to take the binary representations to a proper metric space by simply normalizing the fingerprints. In other words, the transformation:8
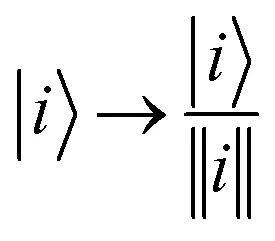

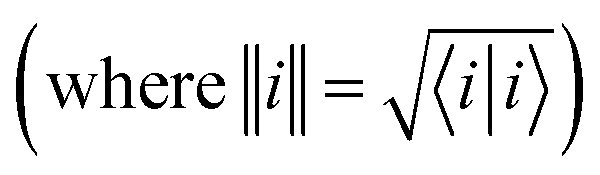
 brings points in the surface of the “binary Tanimoto sphere” to the surface of the “continuous Euclidean sphere”.

Since ultimately our goal is to preserve the local environment as much as possible, we now ask the question: how capable is the *L*_2_ metric over the transformed (normalized) space of capturing the ranking of Tanimoto similarities? To check this, we looked at the relative monotonicity (*e.g.*, consistency) of the Tanimoto and Euclidean values. In more detail: for multiple pairs of molecules, *i* and *j*, we calculate *E*_*ij*_ (the Euclidean distance between molecules) and 1 − *T*_*ij*_. If one of these values increases while the other also increases, both *T* and *E* rank the molecules in the same order. Also, to check the importance of eqn (8), we computed the Euclidean distances over binary and normalized fingerprints.

Fig. 2 shows the relative distribution between the Tanimoto and Euclidian values. As anticipated, the normalized fingerprints do a much better job at preserving the relations from the original binary space. Note the wide spread of values for both ECFP4 and RDKit fingerprints if the Euclidean distance is calculated with binary vectors. On the contrary, the distributions for the normalized vectors are much sharper, indicating a significantly better ranking preservation. As a way to quantify this, we calculated the Kendall correlation coefficients^76^ in all of these cases (see Table 1). Yet again, the utter inadequacy of the binary representation and the vastly superior performance of their normalized continuous counterparts is apparent for both types of fingerprints. It is remarkable that this level of agreement (over 0.9 Kendall correlation) is found just by enforcing perhaps the simplest condition in the chemical space, namely, going from the “Tanimoto sphere” to a “metric sphere”. This leads to the first key conclusion of this contribution: there is much to be gained by utilizing the underlying manifold structure of the chemical space. This has an immediate practical application: if we want to use analysis tools tailored for continuous representations, we do not need to code tailored versions that instead use Tanimoto similarity, we just need to normalize the fingerprints to ensure maximally-preserved relations between neighboring compounds.

**Fig. 2:**
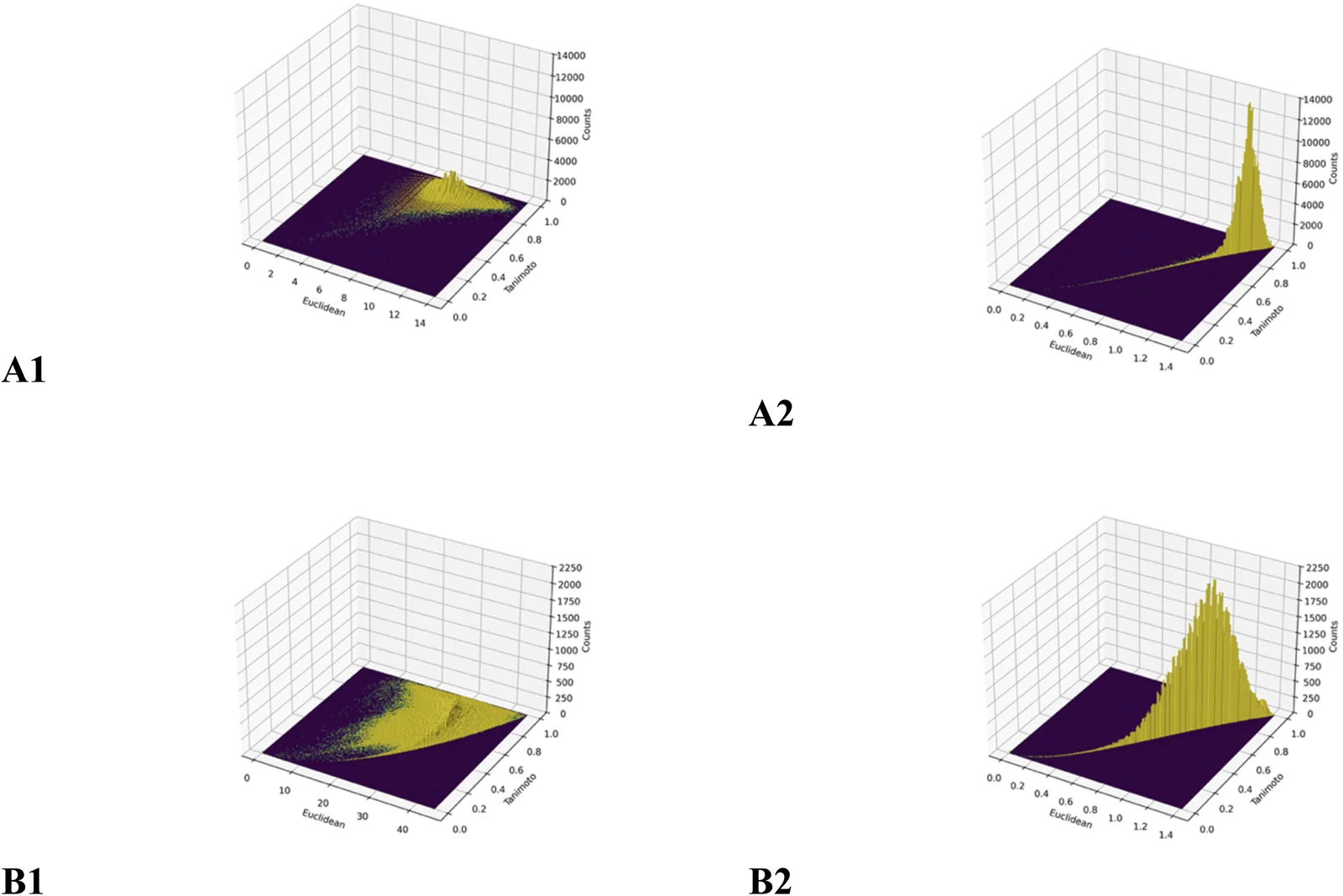
Distribution of Tanimoto and Euclidean values calculated for one million pairs of molecules. ECFP4 (A) and RDKit (B) fingerprints were considered, represented using binary (1) and continuous normalized (2) vectors.

**Table 1:** Kendall's tau values between Tanimoto and Euclidean values for RDKit and ECFP4 fingerprints

	RDKit	ECFP4
Binary fingerprints	0.03	0.07
Normalized vectors	0.91	0.94

### 3.2 Cartography and dimensionality reduction

The results from the previous section paint an enticing picture of a (hyper-)spherical chemical space with molecules resting in its surface. It is natural then to explore how this insight can be used to try to improve the DR results. In particular, we first focus on PCA. To this end, we tested three different protocols:

• Naïve: performing the DR directly from the binary fingerprints.

• Normalized: normalizing the fingerprints, and then performing the DR.

•Projected: normalizing the fingerprints, projecting them into a hyper-plane, and then performing the DR.

In all cases, we chose to project to a two-dimensional space, since this is by far the most popular (and, arguably, convenient) way to visualize chemical space. The naïve method is included as a benchmark, since this is the current approach used in the community. The projected alternative is used to test is an intermediate modification of the chemical space manifold (from spherical to flat) before the DR results in any improvement.

Table 2 shows the scalar metrics for the information lost for the RDKit fingerprints with the 111 projections (the SI has the results for the medoid-centered projections and for the ECFP4 fingerprints). First of all, at the end of the table, we once again corroborate the results presented in the previous section about the great performance of the normalized vectors (before reducing the dimensions to two). Note how just the normalization, or the normalization and then the gnomonic or stereographic projections show markedly high values for all the studied indicators. Notably, while both hyperplane projection methods show essentially identical results for the RDKit fingerprints, the stereographic projection is significantly better than the gnomonic for ECFP4 fingerprints (see the SI section). As expected, however, the distortion caused by the transformation from a spherical to a flat surface, carries an inescapable decrease in quality of the preservation of the local environments, as we can see by comparing the pure stereographic and gnomonic results with the metrics after performing just the normalization.

**Table 2:** Local and global metrics for different combinations of hyper-plane projections, normalization, and PCA

RDKit fingerprints	Dimensions	AUC	*Q*_global_	*Q*_local_	*P*_NN_
PCA (naive)	2	0.63	0.63	0.38	0.09
Norm + PCA (normalized)	2	0.68	0.68	0.39	0.09
Norm + gnomonic 111 + PCA (projected)	2	0.73	0.73	0.39	0.09
Norm + stereographic 111 + PCA (projected)	2	0.69	0.69	0.39	0.10
Norm	2048	0.95	0.95	0.98	0.90
Norm + gnomonic 111	2047	0.86	0.86	0.94	0.77
Norm + stereographic 111	2047	0.89	0.89	0.97	0.84

The results at higher dimensions provide further insights to analyze what happens when we perform PCA to go down to a bi-dimensional representation. Notice how normalizing before PCA provides a slight improvement for all of the considered metrics, as opposed to just doing PCA on top of the original binary fingerprints. Now, however, there is a difference between the hyperplane projections, with the gnomonic results being slightly better. These results are certainly encouraging, but unfortunately they are not as marked for the ECFP4 fingerprints (SI), where the normalization results in 2D are virtually equivalent to those obtained with the original fingerprints. Similar results are observed when the projections are centered around the medoid of the set (see SI).

Fig. 3 shows the results for the other neighborhood preservation indicators. Here, it is easier to see the impact of the normalization. For instance, for the RDKit fingerprints, PCA after normalization provides better results than just doing PCA without normalizing for all descriptors and hyper-plane constructions. This is also the case for ECFP4 fingerprints (see SI), with the exception of the continuity index, which underperforms after normalizing the fingerprints.

**Fig. 3:**
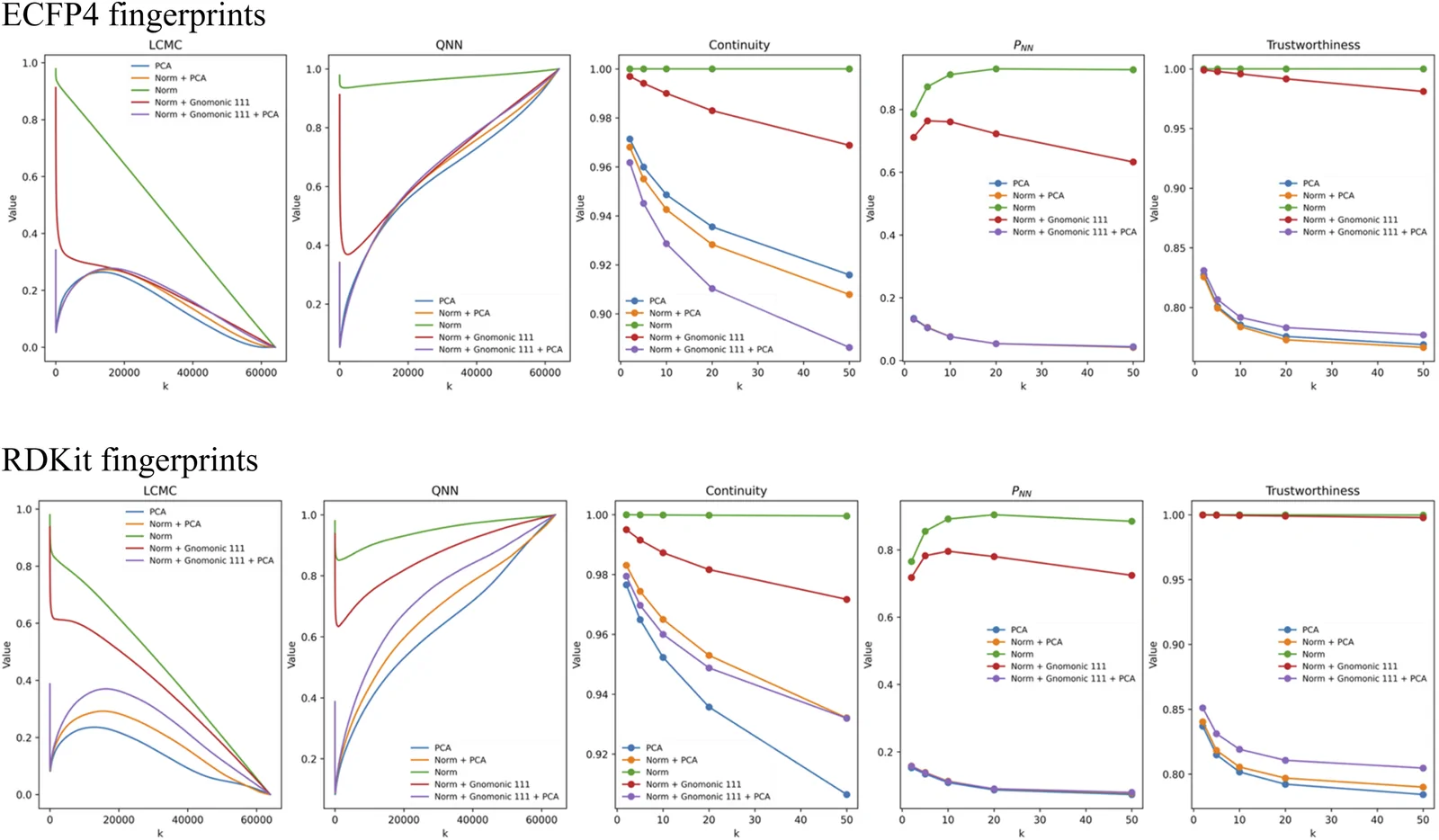
Neighborhood indicators for RDKit and ECFP4 fingerprints for the gnomonic projection.

We evaluated the impact of normalization on non-linear DR methods: t-SNE, UMAP and GTM on CHEMBL219_Ki dataset with 1859 molecules. We already observed noticeable differences in the time it took to process it with the different methods: PCA naturally consumed the least time to project the data, with just 6.5 seconds, while UMAP took about 3 minutes to do the same, and t-SNE and GTM required 19 minutes and 1 hour 10 minutes respectively. Fig. 4 compares some of the neighborhood metrics of the ECFP4 fingerprints of this dataset over the set of hyperparameters (the spread in violin indicates the distribution across hyperparameters) discussed in Section 2.4, which serve to illustrate some key points. The plot of *P*_NN_ (Fig. 4A) reveals that even though non-linear methods are still very sensitive to the choice of initial parameters, they significantly outperform PCA. In particular, t-SNE presents an interesting duality: it is both the best and worst method, with a 70% difference between the optimal and worst hyperparameter set, respectively. Similar results can be observed for the trustworthiness (Fig. 4B) and continuity (Fig. 4C) criteria. Yet again, PCA severely underperforms compared to the non-linear methods, which in turn can provide very good results, if their hyperparameters are carefully chosen. There is, however, another interesting characteristic of the non-linear methods, present both in the global and local metrics, and that is their consistency across the hyperplane projections that we saw could actually improve the PCA results. In other words, neither the normalization nor the hyperplane projections seem to improve the performance of t-SNE, UMAP, or GTM. A plausible explanation for this is that these methods are already extremely flexible, so considering some underlying structure in the high-dimensional fingerprint space does not appreciably impact their outcome. This leads to an obvious question: is it possible to use the insights from the “spherical” distribution of molecules in the high-dimensional space to develop a linear method that could compete with the non-linear projections? The following section responds this in the affirmative.

**Fig. 4:**
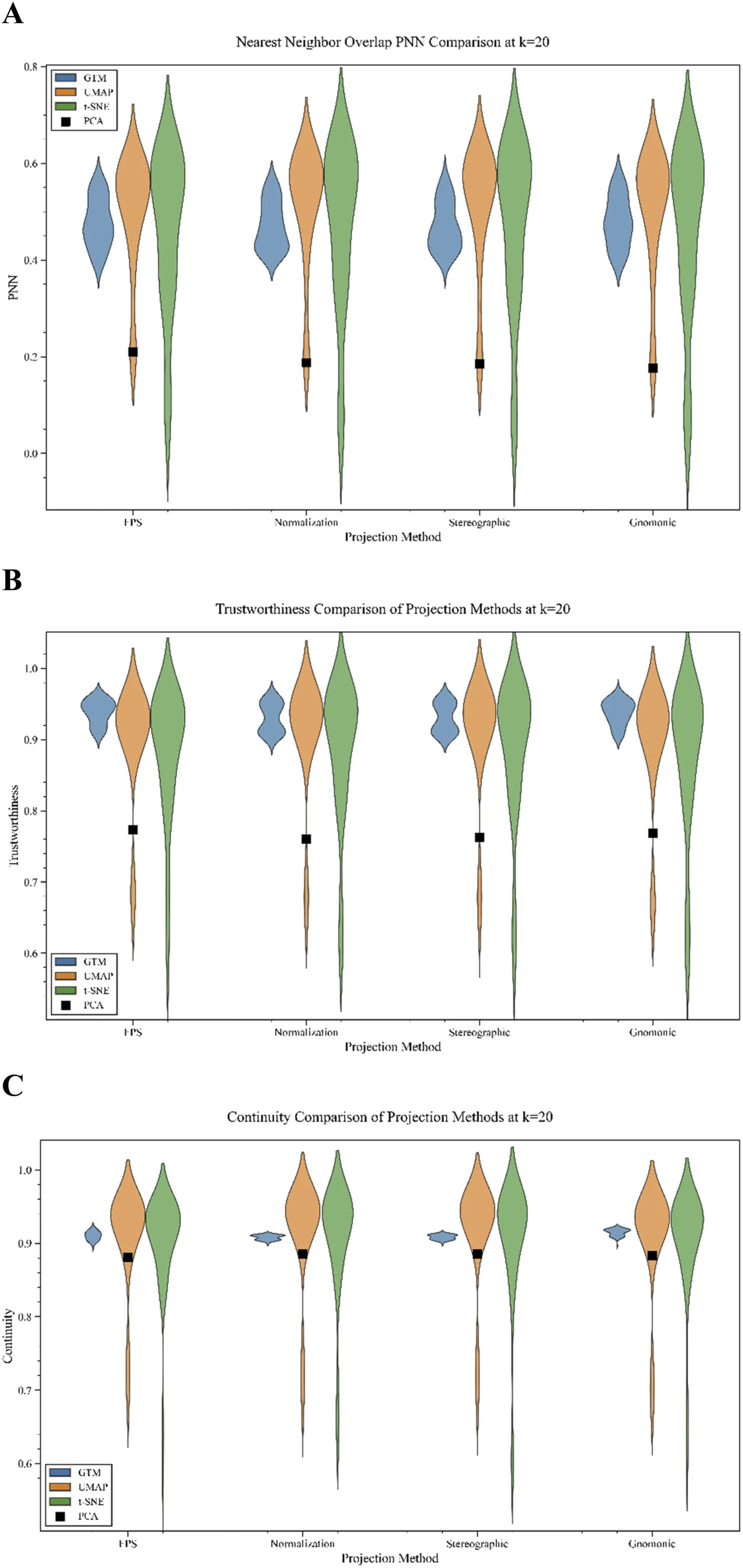
Local metrics for GTM (blue), UMAP (orange), t-SNE (green), and PCA (black) for CHEMBL219_Ki with ECFP4 fingerprints. (A) *P*_NN_, (B) *Q*_local_, (C) trustworthiness, continuity.

### 3.3 Project-after-clustering

The realization that the original fingerprint space endowed with a Tanimoto metric can be put in almost perfect correspondence with a spherical surface with Euclidean distances provides a nice insight into the structure of chemical space, but as noted above, it does not immediately lead to a dramatic improvement in the quality of the projected results. This is easy to rationalize if we remember that the much simpler problem of projecting the surface of our 3D planet into a 2D plane inescapably leads to very distorted regions. Essentially, no matter the nature of the starting (non-linear) manifold, any DR technique will inevitably lose and/or distort information, which is even more pronounced when we go from thousands of dimensions (fingerprints) to just two (not in vain, isometric embedding theorems from non-linear manifolds always require increasing the number of dimensions). However, the potential solution to this issue can be found in how we approach mapping the surface of our planet: while it is possible to have approximate representations of the whole planet, whenever we need extra precision to navigate a given city, we only focus on that portion of the spherical surface, and only project that terrain. Essentially, the smaller the spherical portion that we want to map, the better the approximation of just using a linear (tangential) plane to that section. To apply this insight to chemical spaces, however, we need to find a way to first identify dense (*e.g.* “city-like”) regions in fingerprint space. This calls for a paradigm shift in the relation between clustering and DR: traditionally, DR is performed before the clustering, as a way to reduce the computational cost of the latter. Now, we propose doing the clustering first, as a way to improve the quality and preserve the information of the DR. This project-after-clustering (PAC) recipe also has the advantage of not requiring any hyperparameter tuning, beyond the selection of the neighborhood size for the projection. Of course, this paradigm could be coupled to any DR technique, but it is certainly more attractive to focus on simpler, linear, methods.

In order to establish a baseline to gauge the effects of the clustering approach, in Fig. 5 we “zoom” into the results of PCA and UMAP (done in the traditional way) for the 64k set (we only compare to UMAP because the other non-linear techniques took too long to converge for this library). Yet again, AUC, *Q*_global_, and *Q*_local_ show relatively similar performances for both DR strategies. On the other hand, *P*_NN_ and trustworthiness show a marked improvement for UMAP (when we use the best possible hyperparameters).

**Fig. 5:**
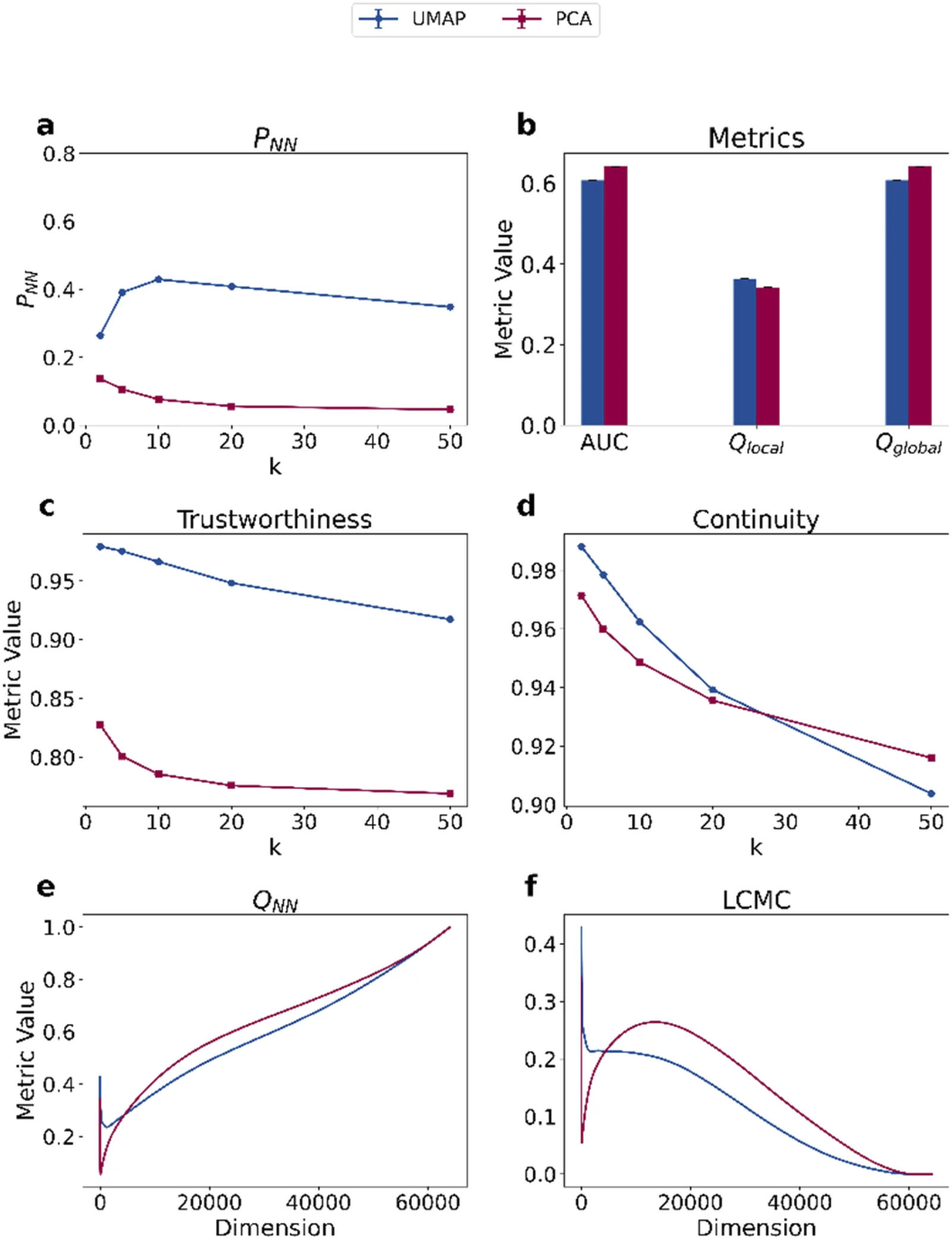
Detailed analysis of neighborhood metrics for the 64k set after projecting using PCA and UMAP for CHEMBL33 with ECFP4 fingerprints.

Fig. 6 presents the same metrics after applying PAC using diameter BitBIRCH clustering (threshold of 0.65 and then a BF refinement, as described in ref. 63), with each cluster projected independently. From this, we can draw two immediate key conclusions: all the metrics improved significantly compared to the analysis of the whole set, and PCA now outperforms UMAP (or, at worst, provides equivalent results). AUC rises from ∼0.6 to near or above 0.8 for both methods, *P*_NN_ values (arguably the most direct indicator of neighborhood fidelity) increase from below 0.2 (full-set PCA) to above 0.4 and approaching 0.8 for BitBIRCH-then-PCA, consistently surpassing UMAP. Trustworthiness and continuity show the expected decline as *k* approaches the cluster size, which was expected as a statistical consequence of evaluating rank-dependent metrics over very small neighborhoods, but PAC-PCA maintains consistent improvement to PAC-UMAP at all granularity levels and fingerprint types (see Fig. S5 and S7).

**Fig. 6:**
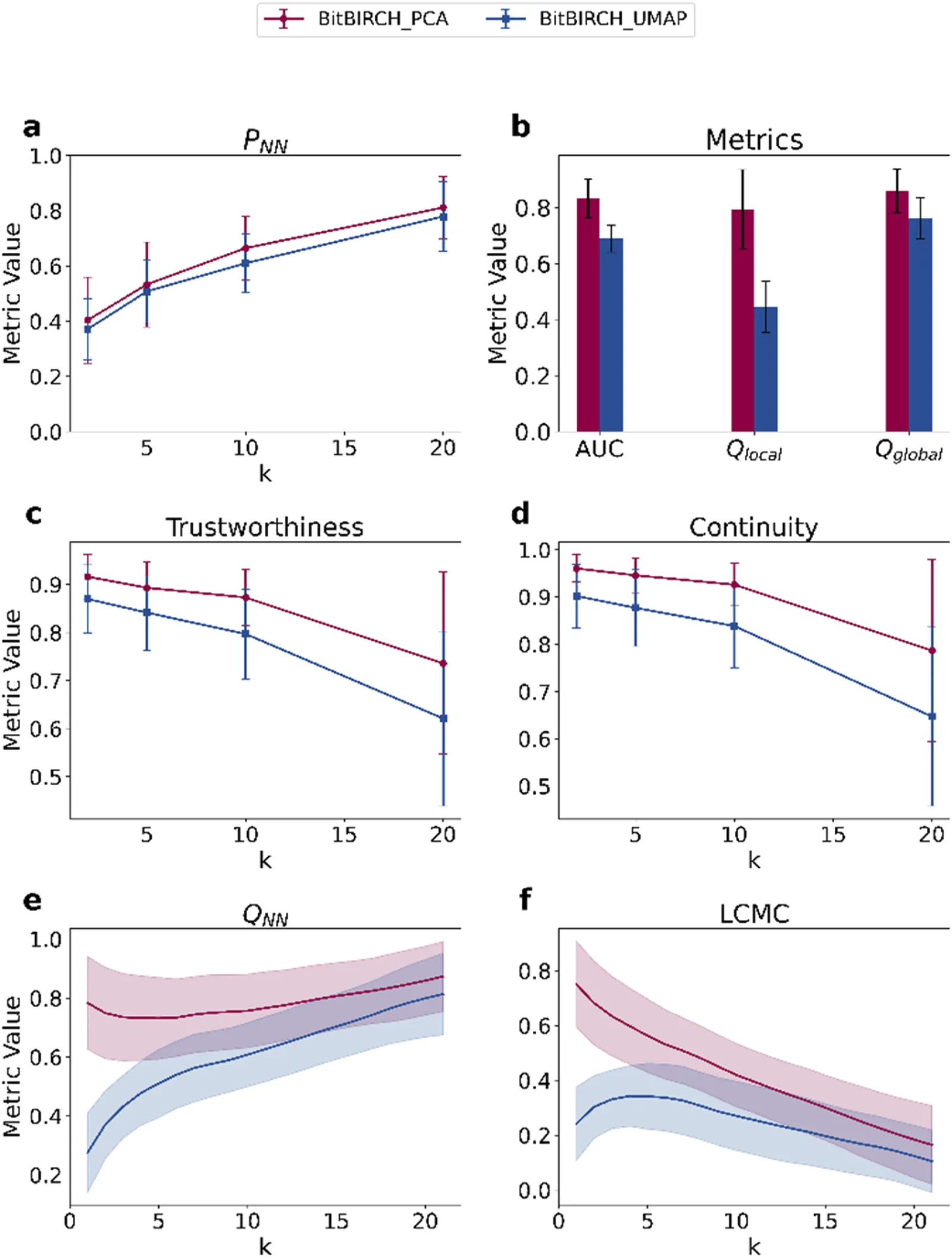
Detailed analysis of neighborhood metrics for the BitBIRCH-then-PCA and BitBIRCH-then-UMAP for clusters having more than 20 molecules.

These results are particularly illuminating confining PCA to local neighborhoods removes the burden of finding an optimal global linear transformation, allowing it to capture local structure with high accuracy. Conversely, reducing the training size of UMAP renders it prone to overfitting, diminishing its performance precisely where it was previously the strongest. The practical advantage is obvious: there is no hyperparameter tuning involved for PAC-PCA, while the performances being at least similar or better than PAC-UMAP with optimal hyperparameters. Add to this that processing all the PCAs for all the clusters only took 30 seconds, while all the UMAPs required 4900 seconds, and the superiority of PCA becomes even more apparent. The procedure thus validates our core hypothesis: analyzing local sections of chemical space provides a dramatically superior strategy for faithful 2D representation, without needing any hyperparameter tuning.

### 3.4 Validation of chemical homogeneity and *post hoc* clustering controls

To rigorously address whether PAC partitions capture genuine chemical structure rather than an arbitrary data decomposition, we evaluated the intra-cluster average Tanimoto similarity (iSIM) and number of unique Bemis–Murcko scaffold families across the top 20 largest clusters produced by three distinct methodologies: (1) BitBIRCH clustering in the native high-dimensional space (the PAC approach), (2) *K*-means clustering applied to the global 2D UMAP projection (at optimal hyperparameter settings), and (3) density-based spatial clustering (DBSCAN) applied to the same global 2D UMAP projection (Fig. 7).

**Fig. 7:**
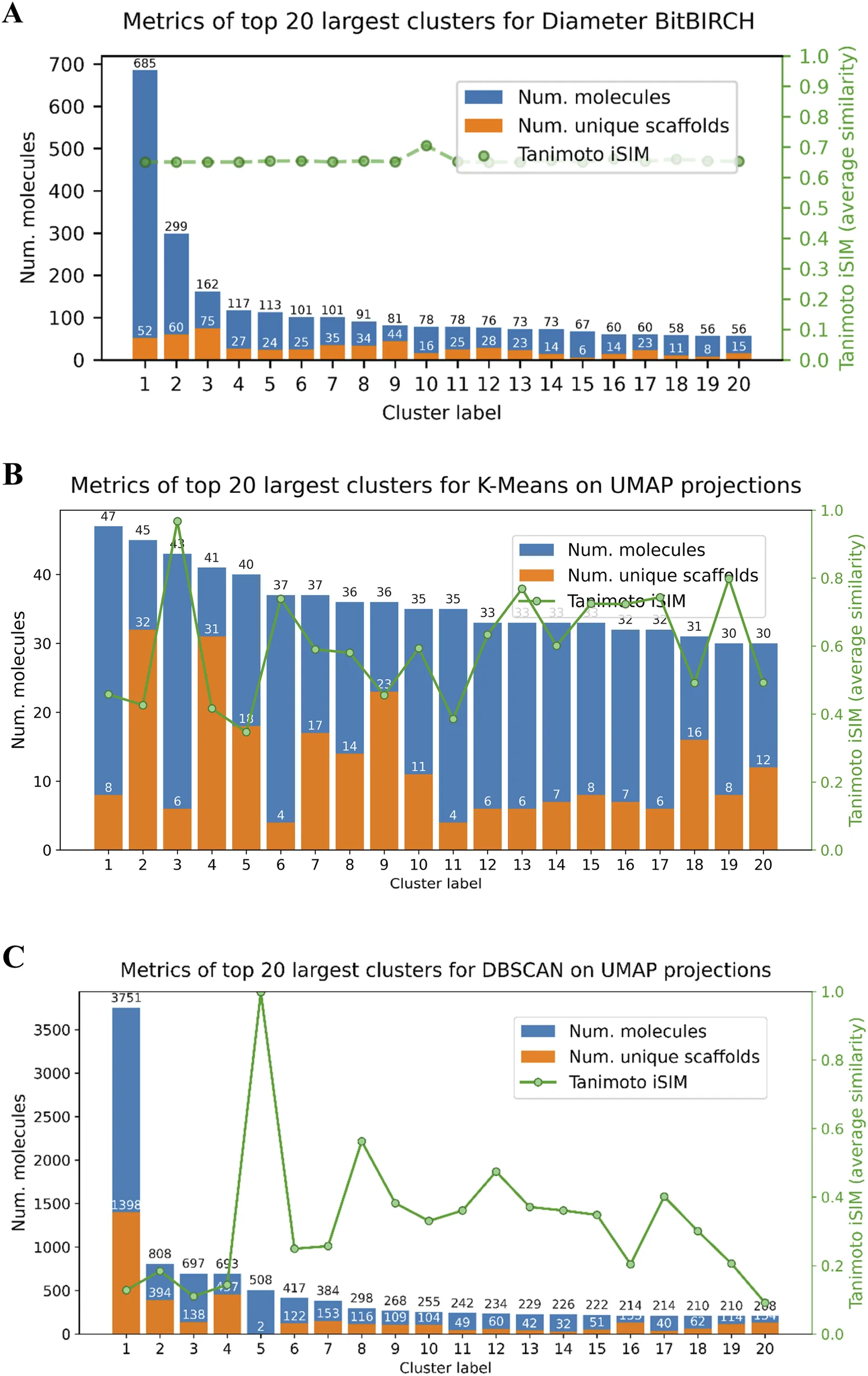
Comparison of scaffold diversity and intra-cluster similarity across the top 20 clusters obtained by (A) BitBIRCH clustering in fingerprint space (PAC procedure) (B) *K*-means clustering applied to the 2D UMAP embeddings and (C) DBSCAN applied to the 2D UMAP embeddings.

#### 3.4.1 Preservation of known chemical classes

Fig. 7A shows results for PAC on the CHEMBL33 dataset containing 64k molecules (overall iSIM = 0.113). Using diameter BitBIRCH with a threshold of 0.65 and branching factor of 1024, we identified 24 579 clusters (14 618 remained as singletons due to the dataset's high diversity). These partitions exhibit remarkable chemical coherence: the average iSIM across all clusters is 0.732 (±0.10), remaining consistently high (∼0.65–0.7) even across the largest clusters, which also demonstrate high scaffold homogeneity (*e.g.*, cluster 1 contains 685 molecules representing only 52 distinct scaffolds). As shown in Fig. S11, increasing the clustering threshold yields even fewer scaffolds per cluster, enabling precise isolation of structural analogs for activity cliff analysis without relying on any external chemical annotations.

#### 3.4.2 Control experiments against partitioning artifacts

There is significant information to be gained about the distribution of molecules in chemical space by clustering the fingerprints before projection in 2D over clustering the 2D projections post-DR, as demonstrated when comparing Fig. 7A–C. To confirm that this chemical fidelity is not an artifact of evaluating smaller subsets, we applied *K*-means to the global UMAP 2D projections, initializing it with *K* = 24 579 to explicitly match the number of BitBIRCH partitions. Forcing this partition upon the 2D embedding fails entirely to recover the underlying chemical structure as the average iSIM drops to 0.593 (±0.242) and clusters become highly heterogeneous. *E.g.*, cluster 4 contains 41 molecules comprising 31 unique scaffolds. This structural mixing is a direct consequence of UMAP's tendency to place structurally dissimilar molecules next to each other when the 2D embedding space is exhausted.

Applying DBSCAN to the same embedding space (Fig. 7C) reveals further distortion. Rather than identifying distinct chemical series, DBSCAN isolates a single, massive, continuous dense region inherent to the crowding problem of non-linear DR. Even with parameter tuning, the resulting partitions remain chemically meaningless: the largest cluster absorbs ∼3700 molecules encompassing ∼1400 unique scaffold families with iSIM <0.2, confirming that the 2D embeddings collapse distinct chemical series into an indistinguishable spatial region.

#### 3.4.3 Methodological robustness and hyperparameter independence

Collectively, these experiments demonstrate that PAC's chemical homogeneity is a consequence of the methodological workflow itself, rather than data subdivision. The critical difference is where the partition occurs: in the undistorted fingerprint space where molecular similarity relationships are preserved exactly, rather than in a compressed 2D space where they have already been irreversibly distorted. Extracting chemical series *post hoc* additionally creates a cascading hyperparameter problem: non-linear methods like UMAP are sensitive to hyperparameters like n_neighbors and min_dist and secondary density-based clustering further introduces more hyperparameters, yielding a two-stage optimization problem which is computationally prohibitive, subjective and ultimately futile.

In contrast, the PAC framework overcomes this dependency chain entirely. By performing partitioning directly in the high-dimensional space, where Tanimoto distances are invariant to embedding parameters, PAC eliminates the need for any cascading hyperparameter tuning. The user is only required to define the initial clustering similarity threshold based on their specific objective: a general chemical space mapping can be achieved at a threshold of 0.6–0.7, whereas identifying highly similar analogs with a low scaffold-to-molecule ratio can be achieved at thresholds >0.85. The resulting partitions are deterministic, reproducible, and most importantly chemically meaningful.

### 3.5 N-Ary mapping interface

With the insights obtained in previous sections, we built the N-Ary Mapping Interface (NAMI), a visualization pipeline providing a two-level view of chemical space. It is implemented as an open-source GUI, freely available at https://github.com/mqcomplab/NAMI. To benchmark NAMI at scale, we mapped the ZINC20 dataset containing over 10 million molecules using 30 Intel Xeon Platinum 8570 CPU cores. The entire pipeline, from SMILES loading through fingerprint generation, BBlean clustering to global centroid PCA completed in under 2.5 minutes with just 7 GB of RAM (Fig. 8 shows an illustration of the user interface at the global and cluster level). We also investigated the faithfulness of the workflow by performing clustering at a threshold of 0.65 and branching factor of 1024 on the ZINC20 dataset, consuming 15.8 GB of memory to complete the process in under 8 minutes. As shown in Fig. 9, the 20 largest clusters contain between 618 and 1181 molecules each, with intra-cluster iSIM values consistently maintained at 0.65–0.7 across all clusters, a uniform behavior which consistently scales to ultra-large chemical libraries that stands in stark contrast to the erratic behavior of the non-linear projections in the previous sections.

**Fig. 8:**
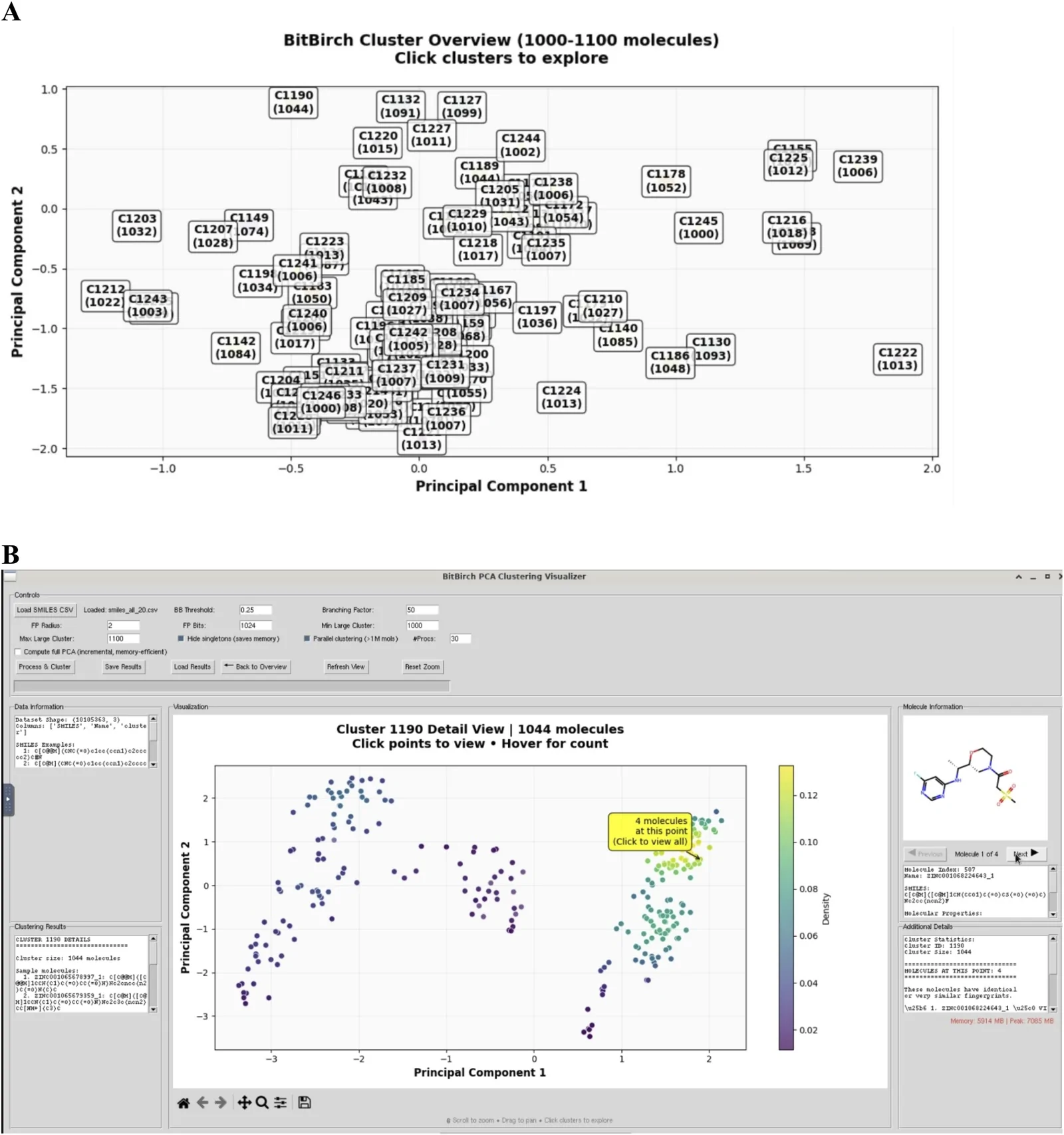
Sample visualization with NAMI of the 10m library selected from ZINC20. (A) Distribution of clusters containing in between 1000 to 1100 molecules, projected from the cluster centroids. (B) Local visualization of a cluster.

**Fig. 9:**
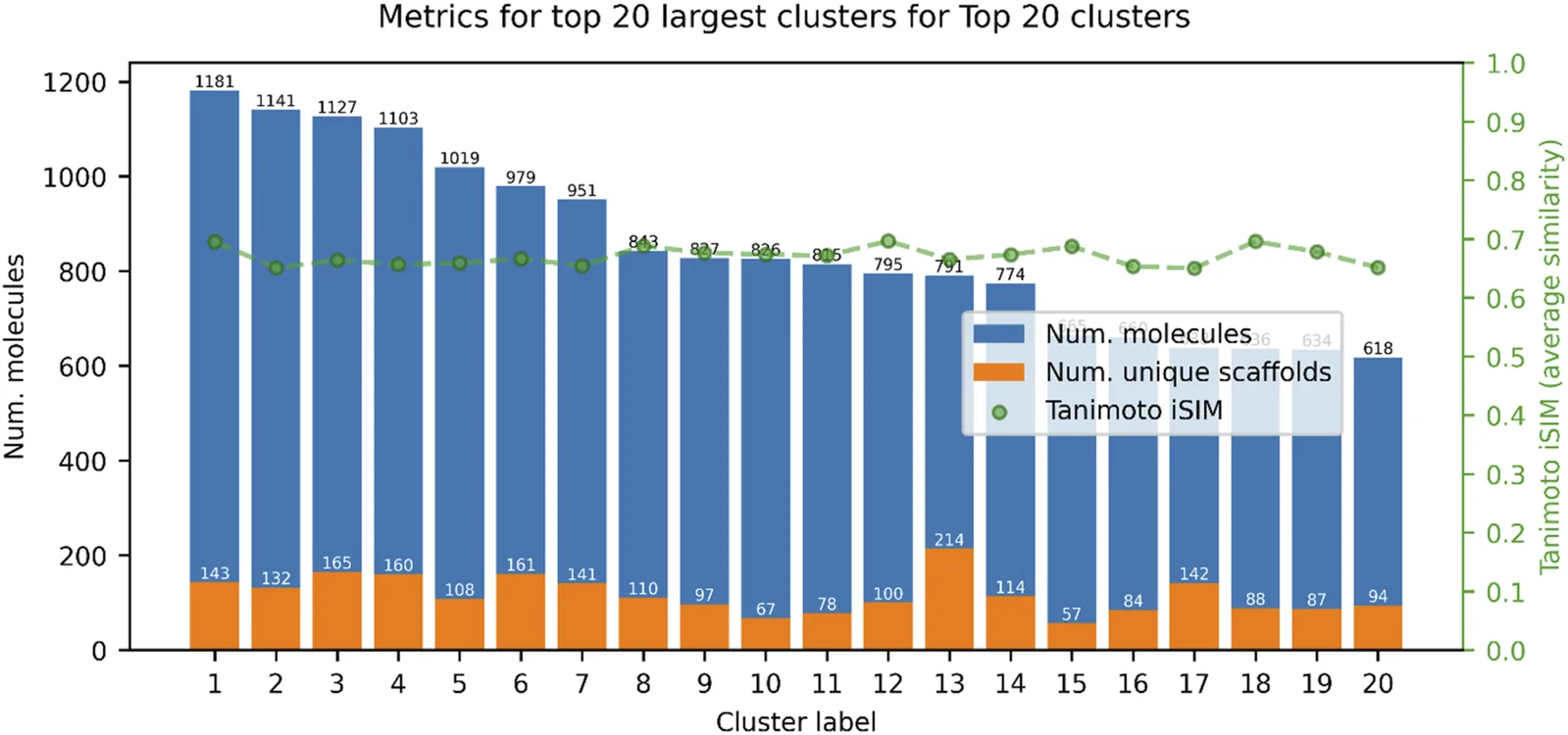
Scaffold diversity and intra-cluster similarity across the top 20 clusters obtained by performing NAMI on the ZINC20 10 million molecular dataset.

Inspection of the most frequent Bemis–Murcko scaffolds within representative clusters further confirms their chemical coherence (Fig. 10). In each case, the top 5 dominant scaffolds occupy between 27% to 52% of the cluster population, indicating that the clusters are simultaneously chemically focused (dominated by a small number of related core scaffolds). Given the similarity between the top 5 scaffolds themselves, which is very evident in Fig. 10C, this is worth stating that the clustering algorithm is grouping compounds by shared molecular structure with majority of the molecules sharing a wide range of highly similar scaffolds. While just a proof-of-principle implementation in its current state, NAMI already provides a faithful representation of chemical space relying only on very efficient tools (bblean and PCA), by focusing on the locally dense sectors of a library and avoiding the tuning and overfitting of non-linear DR methods.

**Fig. 10:**
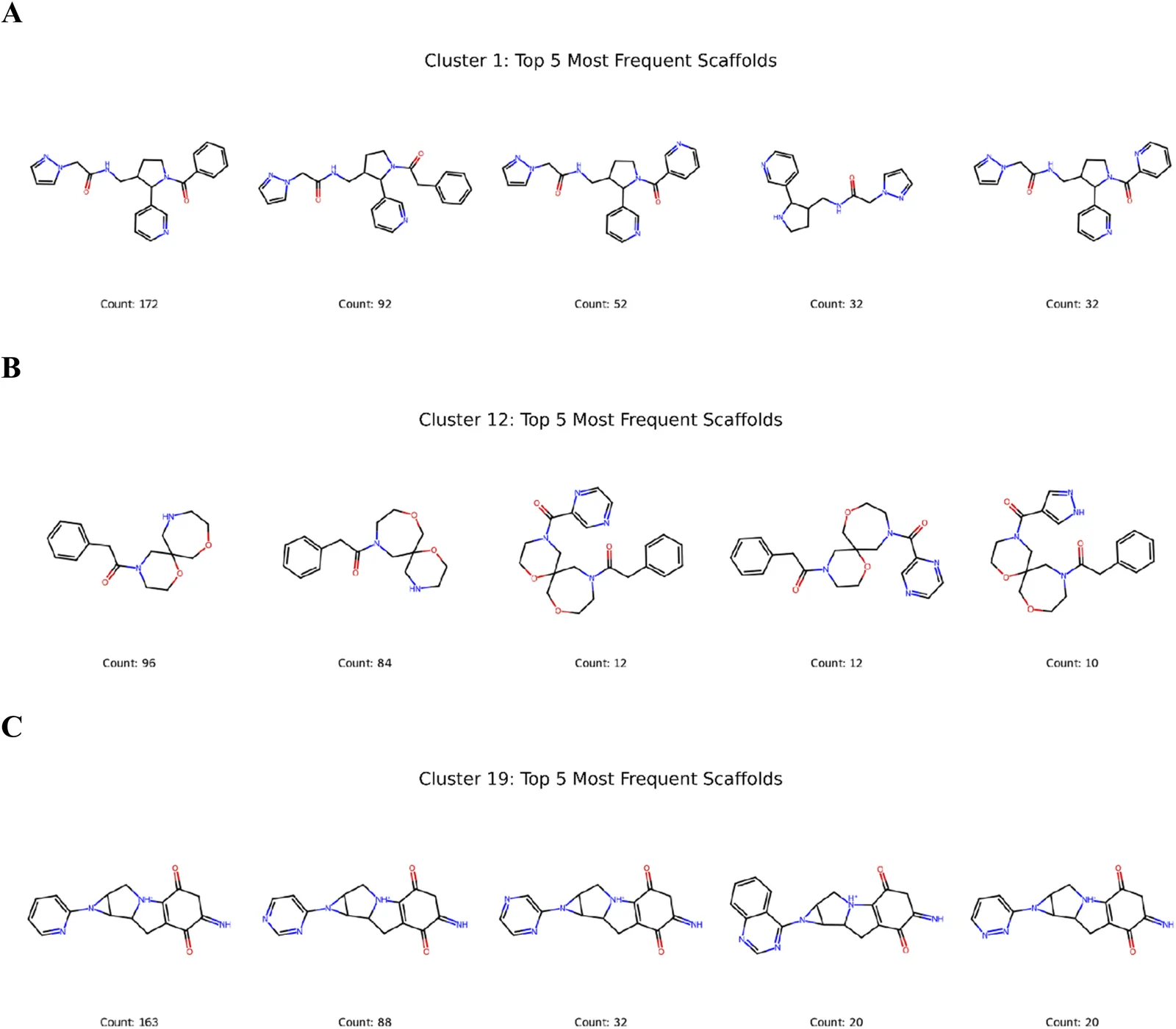
Top 5 Bemis–Murcko scaffolds for cluster 1, 12 and 19 of the ZINC20 dataset obtained after performing NAMI.

## 4 Conclusions

We have demonstrated that linear dimensionality reduction can match or even surpass non-linear alternatives in both speed and neighborhood preservation when the inherent topology of fingerprint space is properly exploited. The central theoretical result is that binary fingerprints under the Tanimoto metric reside on a hyperspherical surface and that normalizing the fingerprints to a continuous Euclidean space preserves pairwise similarity rankings with excellent fidelity, and the resulting smooth sections can be faithfully described by simple linear tools, making flexible non-linear methods unnecessary at the cluster level. Taking inspiration from cartography, the PAC pipeline inverts the conventional paradigm between clustering and dimensionality reduction, rather than using clustering to interpret the results of dimensionality reduction, PAC uses clustering to enhance the quality of dimensionality reduction. The reason for this being that clustering in the high-dimensional fingerprint space helps preserve the similarity relationships which are inevitably distorted when projecting the entire dataset using non-linear dimensionality reduction methods. This strategy elevates PCA to performance levels on par with or superior to hyperparameter-optimized UMAP or t-SNE for local neighborhood preservation, at over 160-fold lower computational cost for libraries and scaling to ten million compounds in under 2.5 minutes and about 7 GB of RAM, hence transforming what previously needed days of compute and terabytes of memory into a routine workstation task *via* the NAMI implementation.

Beyond neighborhood preservation metrics, PAC partitions carry genuine chemical meaning. Analysis of Bemis–Murcko scaffold diversity across CHEMBL33 and ZINC20 datasets reveals high scaffold homogeneity within clusters, confirming that BitBIRCH clustering in the fingerprint space successfully identifies coherent chemical series without the need of any external annotations. Strict control experiments by applying *K*-means and DBSCAN *post hoc* to UMAP projections with the same number of partitions yield chemically heterogeneous clusters while displaying chaotic behavior in intra-cluster similarities, demonstrating that the spatial crowding inherent to non-linear dimensionality reduction methods inevitably destroys the structure that PAC preserves. These chemically coherent partitions are directly actionable; each cluster constitutes a navigable analog series which chemists can use to study structure–activity relationships and prioritize compounds for synthesis, establishing NAMI as a practical tool for scalable, chemically interpretable visualization of large molecular libraries.

Several limitations of the NAMI framework must be acknowledged. First, the theoretical foundation is restricted to binary fingerprints evaluated under the Tanimoto metric. The hyperspherical manifold property that justifies continuous normalization does not extend to continuous descriptors or graph-based molecular representations. Second, as an inherent consequence of the two-step projection paradigm, global spatial relationships between molecules in two different clusters are not well represented, since each partition occupies an independent 2D coordinate system. Finally, in highly structurally diverse libraries, molecules that do not satisfy the clustering similarity conditions are assigned as singletons and do not actually benefit from the projection framework. Users working with highly diverse collections may mitigate this by adopting lower similarity thresholds at the cost of less homogeneous partitions.

## Author contributions

AS: conceptualization, methodology, software, validation, formal analysis, investigation, data curation, writing – original draft, writing – review & editing, visualization. KZ: conceptualization, methodology, software, validation, formal analysis, investigation, data curation, writing – original draft, writing – review & editing, visualization. LC: validation, formal analysis, investigation, data curation, writing – review & editing. RAMQ: conceptualization, methodology, software, formal analysis, investigation, resources, writing – original draft, writing – review & editing, supervision, project administration, funding acquisition.

## Conflicts of interest

The authors have no conflict of interests.

## Supplementary Material

SC-017-D6SC03716J-s001

## Data Availability

The data and code supporting the findings of this study are freely available at https://github.com/mqcomplab/NAMI. Supplementary information (SI) is available. See DOI: https://doi.org/10.1039/d6sc03716j.
