# Supplementary material for: Efficient visualization of chemical space
Source: Chem Sci. 2026 Sep 11. Online ahead of print. doi: 10.1039/d6sc03716j (PMC13583449; doi:10.1039/d6sc03716j)
Supplement: Supplementary file 1 [file SC-OLF-D6SC03716J-s001.pdf]

Supplementary Information (SI) for Chemical Science.
This journal is © The Royal Society of Chemistry 2026

Supplementary Information: Efficient Visualization of Chemical Space

Akash Surendran,[1†*] Krisztina Zsigmond,[1†] Lexin Chen,[1] Ramón Alain Miranda-Quintana[1*]

Department of Chemistry and Quantum Theory Project, University of Florida, FL, Gainesville, 32611

[†]: These authors contributed equally

Email: quintana@chem.ufl.edu, ak.surendran@ufl.edu

S1 Dimensionality reduction after projecting the hypersphere

The scalar neighborhood preservation metrics for the ECFP4 fingerprints with the 111 projections are listed in Table S1. We can observe the same trend in the metrics as for the RDKit fingerprints in Table 2. Normalizing the fingerprints preserves the most amount of neighborhood information, while performing PCA to get to two dimensions from the original 1024 dramatically worsens it.

In addition to the projections with the 111 vector, we performed the neighborhood analysis for the medoid projections (see Tables S2 and S3). This change in the projection vector does not lead to significantly different results.

Table S1: Neighborhood information for the ECFP4 fingerprints with the 111 projections.

ECFP4	Dimensions	AUC	Q_{global}	Q_{local}	k_{max}	P_{NN}
PCA (Naïve)	2	0.64	0.64	0.34	1	5.38
Norm + PCA (Normalized)	2	0.66	0.66	0.34	1	5.37
Norm + Gnomonic 111 + PCA (Projected)	2	0.67	0.67	0.34	1	5.40
Norm + Stereographic 111 + PCA (Projected)	2	0.66	0.66	0.34	1	5.32
Norm	1024	0.97	0.97	0.98	1	92.92
Norm + Gnomonic 111	1023	0.69	0.69	0.91	1	72.26
Norm + Stereographic 111	1023	0.89	0.89	0.97	1	91.82

Table S2: Neighborhood preservation metrics for the ECFP4 fingerprints. Here we are using the medoid projections.

ECFP4	Dimensions	AUC	Q_{global}	Q_{local}	k_{max}	P_{NN}
PCA (Naïve)	2	0.64	0.64	0.34	1	5.38
Norm + PCA (Normalized)	2	0.66	0.67	0.34	1	5.37

Norm + Gnomonic medoid + PCA (Projected)	2	0.66	0.66	0.35	1	5.63
Norm + Stereographic medoid + PCA (Projected)	2	0.66	0.66	0.34	1	5.41
Norm	1024	0.97	0.97	0.98	1	92.92
Norm + Gnomonic medoid	1023	0.70	0.70	0.87	1	67.92
Norm + Stereographic medoid	1023	0.85	0.85	0.94	1	83.30

Table S3: Neighborhood information loss for the RDKit fingerprints with the medoid projections.

RDKit fingerprints	Dimensions	AUC	Q_{global}	Q_{local}	k_{max}	P_{NN}
PCA (Naïve)	2	0.63	0.63	0.38	1	8.66
Norm + PCA (Normalized)	2	0.68	0.68	0.38	1	8.94
Norm + Gnomonic medoid + PCA (Projected)	2	0.73	0.73	0.39	1	8.99
Norm + Stereographic medoid + PCA (Projected)	2	0.69	0.69	0.39	1	9.81
Norm	2048	0.95	0.95	0.98	1	90.41
Norm + Gnomonic medoid	2047	0.86	0.87	0.94	1	78.01
Norm + Stereographic medoid	2047	0.89	0.89	0.97	1	83.70

The remaining neighborhood preservation metrics, like LCMC and continuity, are depicted in Figures S1-S3. The choice of the projection vector does not affect the results significantly. Furthermore, PCA is outperformed by the projection methods for the RDKit fingerprints. Meanwhile, the naïve PCA approach outperforms the projections only for the continuity metric for the ECFP4 fingerprints. These trends are in agreement with the results for the gnomonic 111 projection from the paper.

ECFP4 fingerprints

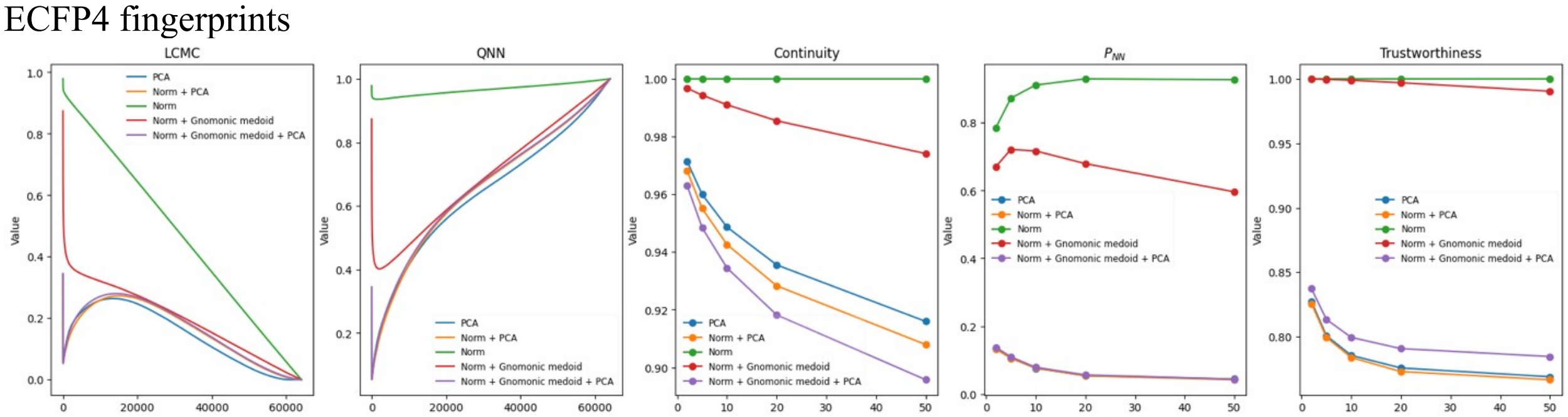

RDKit fingerprints

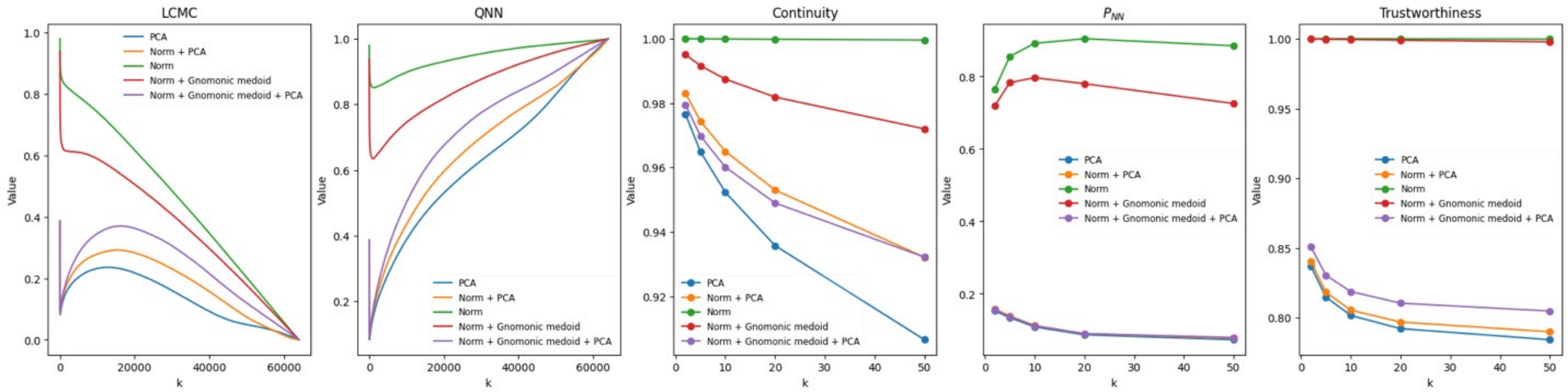

Figure S1: Neighborhood preservation metrics for the gnomonic medoid projection

ECFP4 fingerprints

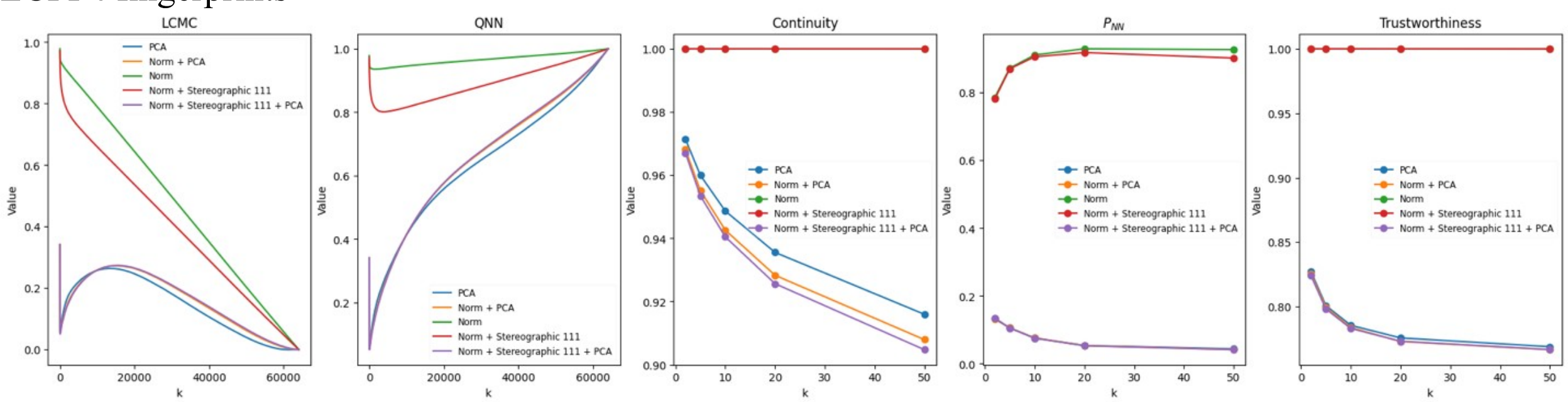

RDKit fingerprints

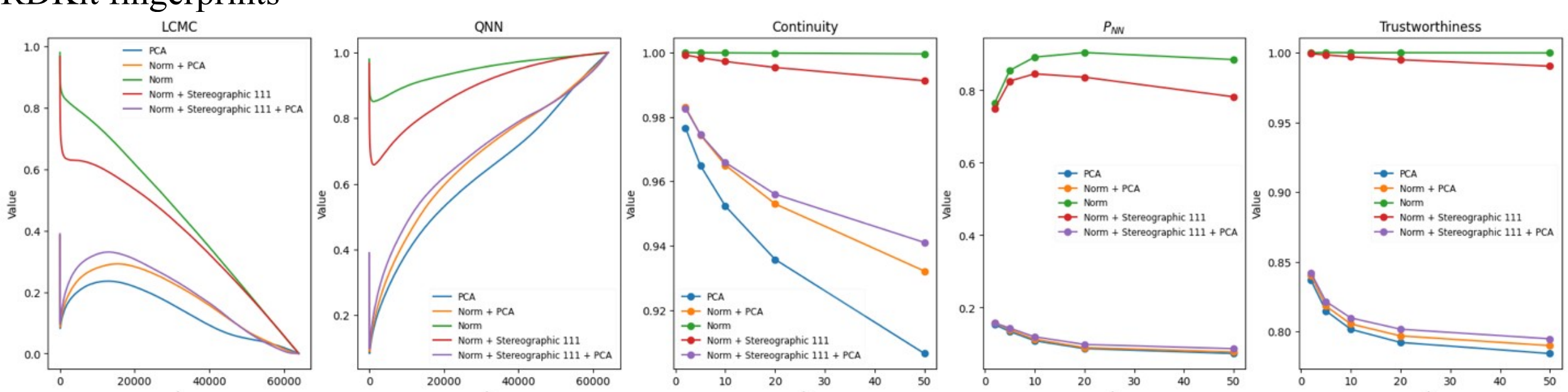

Figure S2: Neighborhood preservation metrics for the stereographic 111 projection

ECFP4 fingerprints

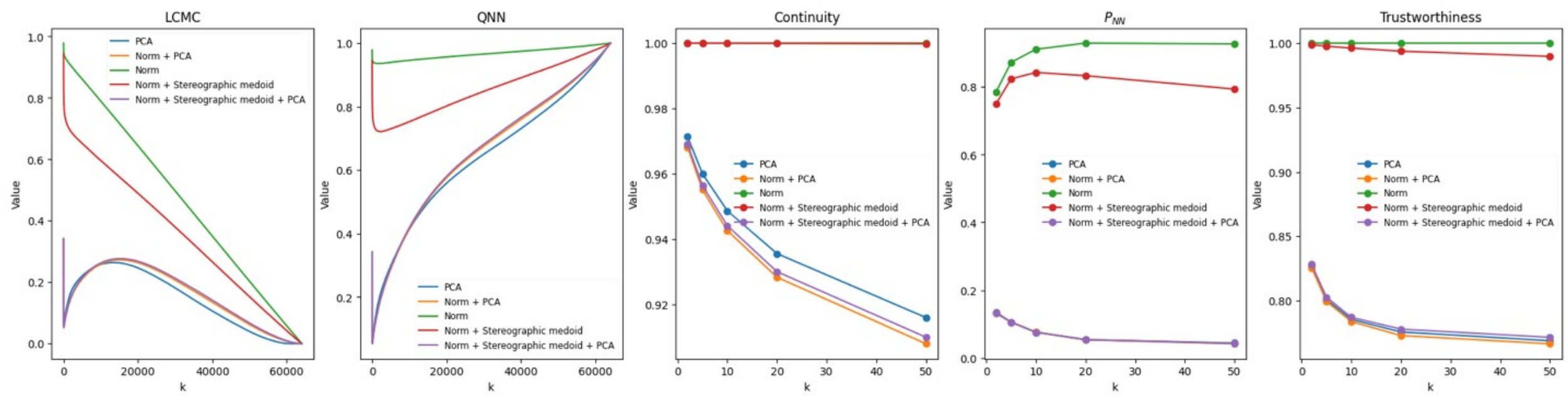

RDKit fingerprints

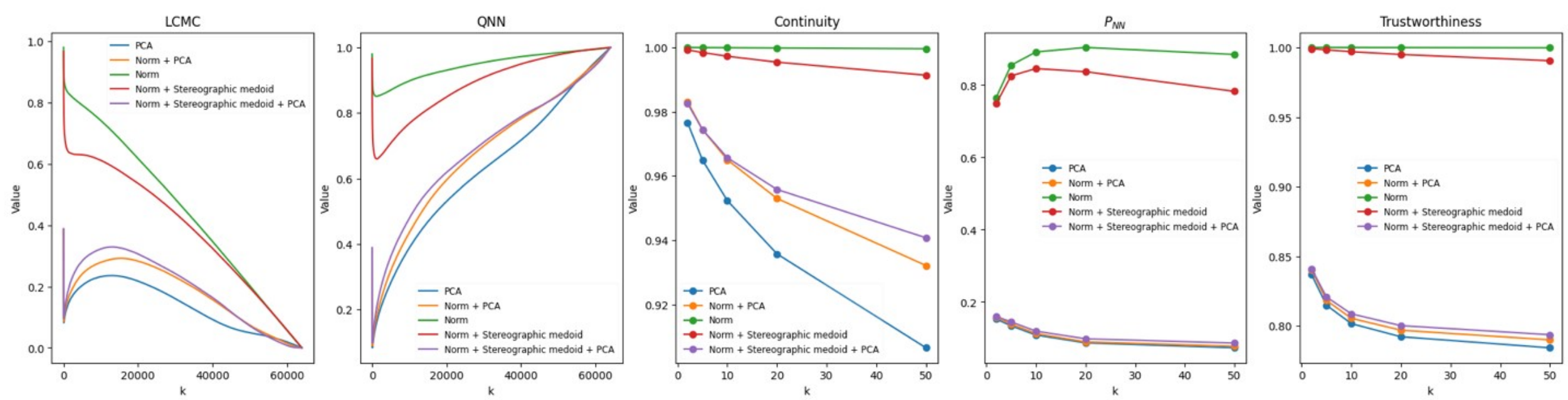

Figure S3: Neighborhood preservation metrics for the stereographic medoid projection

S2. Global neighborhood metrics for CHEMB219_Ki dataset

A

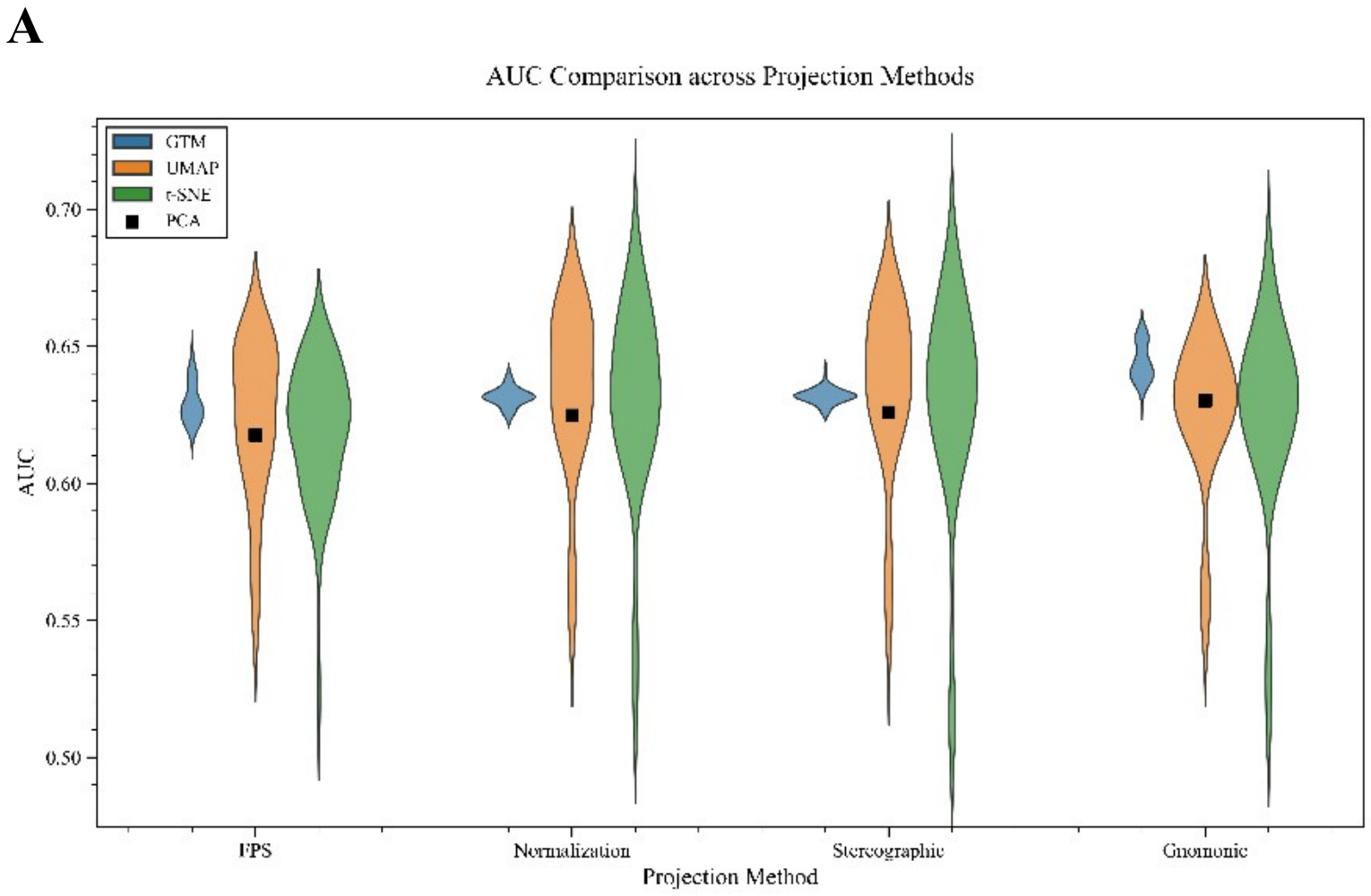

B

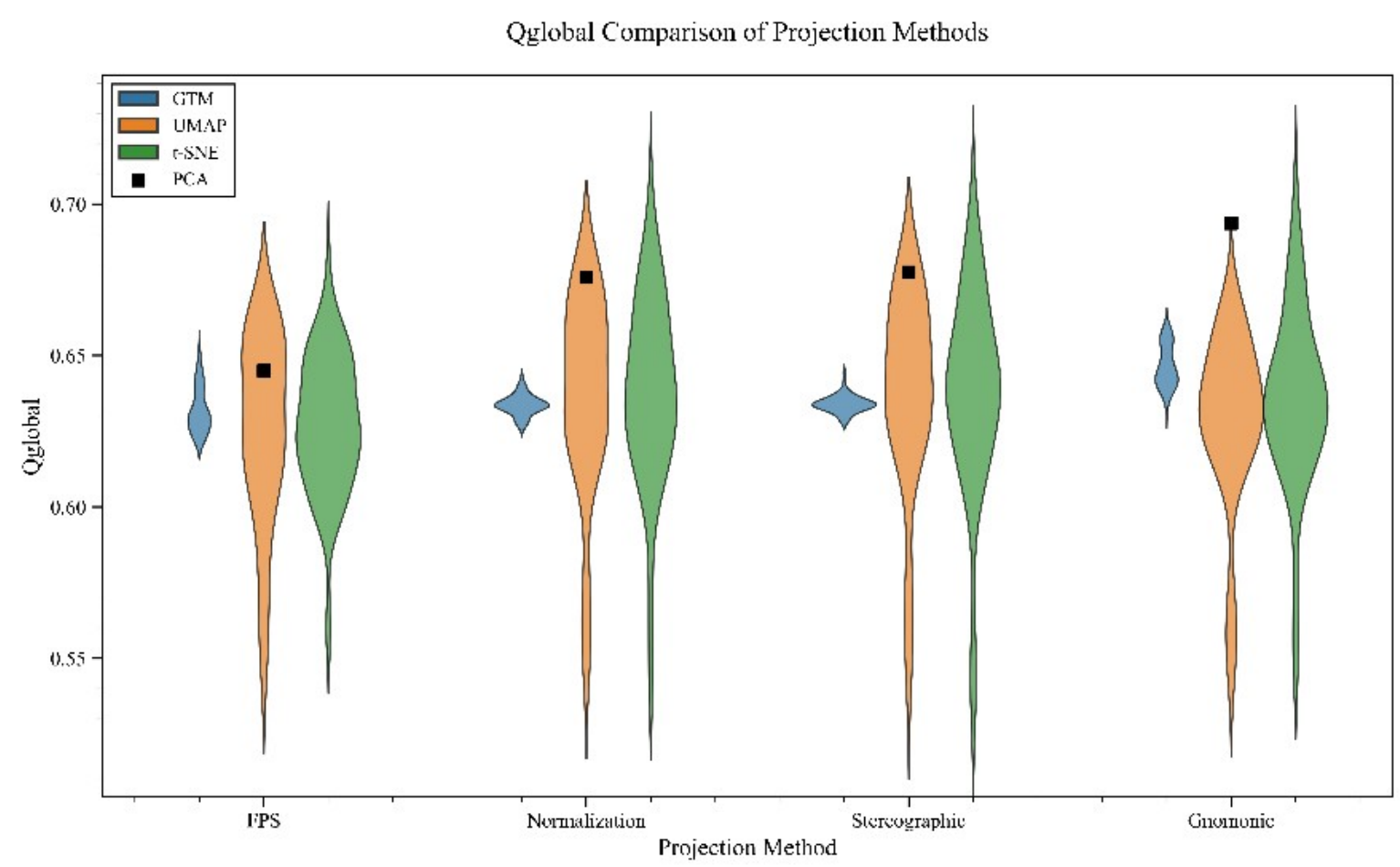

Figure S4: Global metrics for GTM (blue), UMAP (orange), t-SNE (green), and PCA (black) for CHEMBL219_Ki with ECFP4 fingerprints. A: AUC, B: Q_{global}.

Figure S4 compares the global neighborhood preservation metrics for CHEMBL219_Ki across the set of dimensionality reduction method and violin spread across hyperparameters. For AUC (Fig. 4A), the average performance is consistent across all DR methods over all types of projections and shows similar values on average. All the non-linear methods just marginally outperform PCA in their best settings. GTM is the most stable across the hyperparameter set, showing a slight deviation (less than 0.04) across the range, while the performance of t-SNE and UMAP shows larger variations. Similar consistency in values can be observed for the case of Q_{global} as well (Fig. 4B) AUC and Q_{global} show similar violin shapes indicating a small k value for the inflection point (k_{max}) of LCMC curve, hinting at a small Q_{local} contribution to the Q_{NN} curve.

S2. BitBIRCH then PCA/UMAP for ECFP4 fingerprints

Complete analysis for clusters containing more than 10 and 50 molecules.

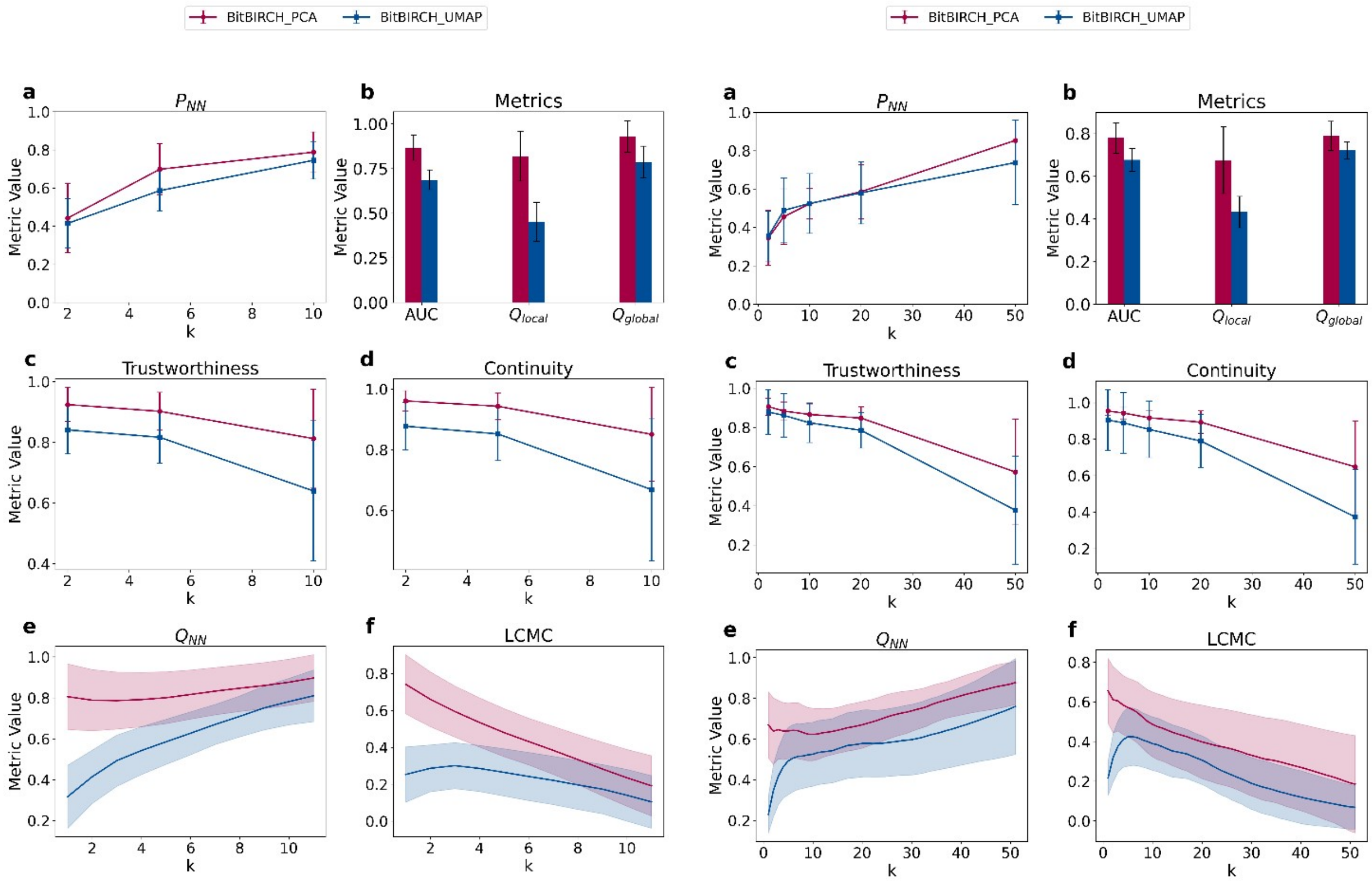

Figure S5: Detailed analysis of neighborhood metrics for the BitBIRCH-then-PCA and BitBIRCH-then-UMAP for ECFP4 fingerprint clusters having more than left: 10 and right: 50 molecules.

Figure S5 shows the results for neighborhood preservation metrics for the BitBIRCH-then-PCA and BitBIRCH-then-UMAP paradigms applied to ECFP4 fingerprint clusters. Consistent with the results for clusters with more than 20 molecules in the main text, the local neighborhood preservation metric P_{NN} remains nearly identical between both approaches, while rank based metrics like Trustworthiness and Continuity in BitBIRCH-then-PCA slightly overperform those in BitBIRCH-then-UMAP. Notably, a steeper decline is observed for these metrics at higher neighborhood sizes (large k), indicating higher rank discrepancies when broader neighborhoods are considered. The results are consistent across all minimum cluster sizes, and the following section deals with how the choice of an alternative fingerprint representation could potentially influence the quality of dimensionality reduction.

S3. Comparison of dimensionality reduction metrics for RDKit fingerprints

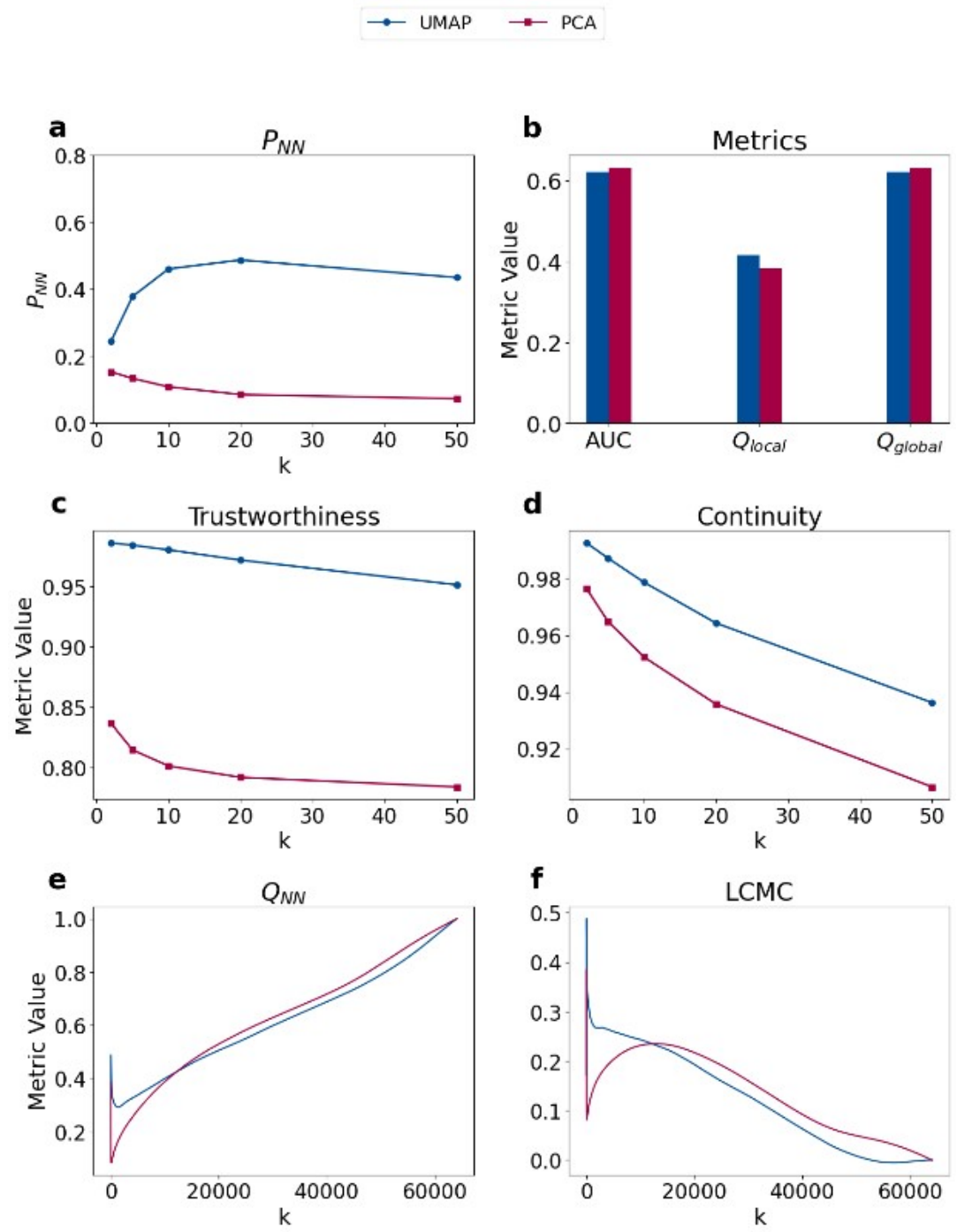

Figure S6: Detailed analysis of neighborhood metrics for the 64K set after projecting using PCA and UMAP for the 2048-dimensional RDKit fingerprints.

An analogous analysis to Figure 5 of the main text, now utilizing 2048-dimensional RDKit fingerprints instead of ECFP4 shows little to no differences to the results of ECFP4, indicating that these dimensionality reduction methods perform consistently regardless of the dimensionality of the fingerprint vector (1024 or 2048). As expected, local neighborhood preservation metrics like P_{NN}, Trustworthiness and Continuity portray a superior performance for UMAP to PCA.

We performed the cluster-then-project procedure for RDKit fingerprints to check for consistency in the improvement of projection metrics across molecular representations. Figure S7 presents the results for clusters containing a minimum of 11, 21 and 51 molecules, respectively. As before, we performed diameter BitBIRCH with a 0.65 similarity threshold and BF refinement. The results are again consistent to the one in the main text, with BitBIRCH-PCA consistently delivering reliable projections for yet another fingerprint type. Local metrics like P_{NN}, Trustworthiness and Continuity show better performance across all k values for all cluster size settings for BitBIRCH-PCA, aligning with the trends observed for ECFP4 fingerprints.

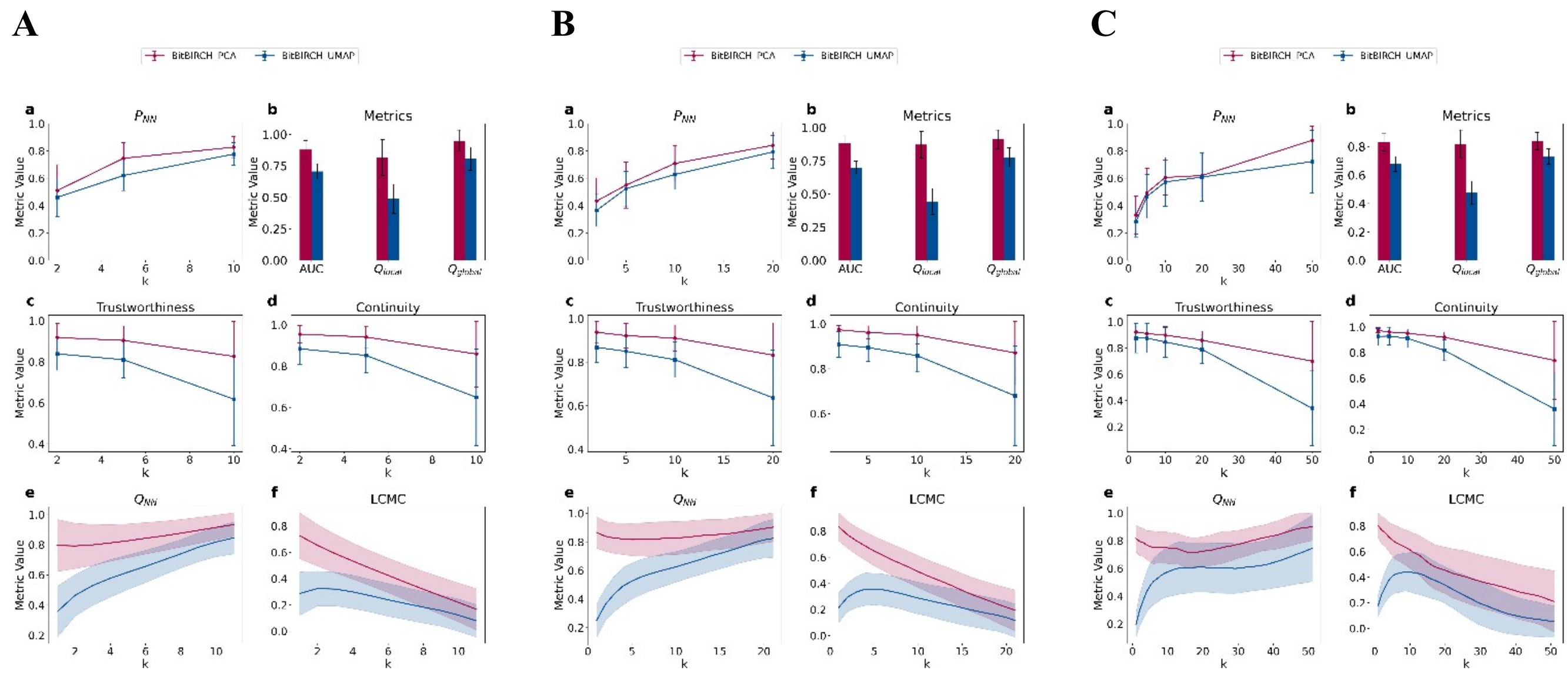

Figure S7: Detailed analysis of neighborhood metrics for the BitBIRCH-then-PCA and BitBIRCH-then-UMAP on RDKit fingerprints for clusters having at least A) 11, B) 21 and C) 51 molecules.

S4. Comparison of DR metrics across Normalized and Gnomonic centroid projections

Building upon the cartography results for PCA, we attempt to see if performing these projections before the BitBIRCH-then-PCA recipe provides any profound insights. The results for BitBIRCH-PCA performed on Normalized fingerprints and Gnomonic centroid projected fingerprints are shown in Figure S7 and S8 respectively. The metric values and trends appear closely similar to those in the plain ECFP4 case, indicating that projecting prior to dimensionality reduction offers little to no change in the quality of neighborhood preservation.

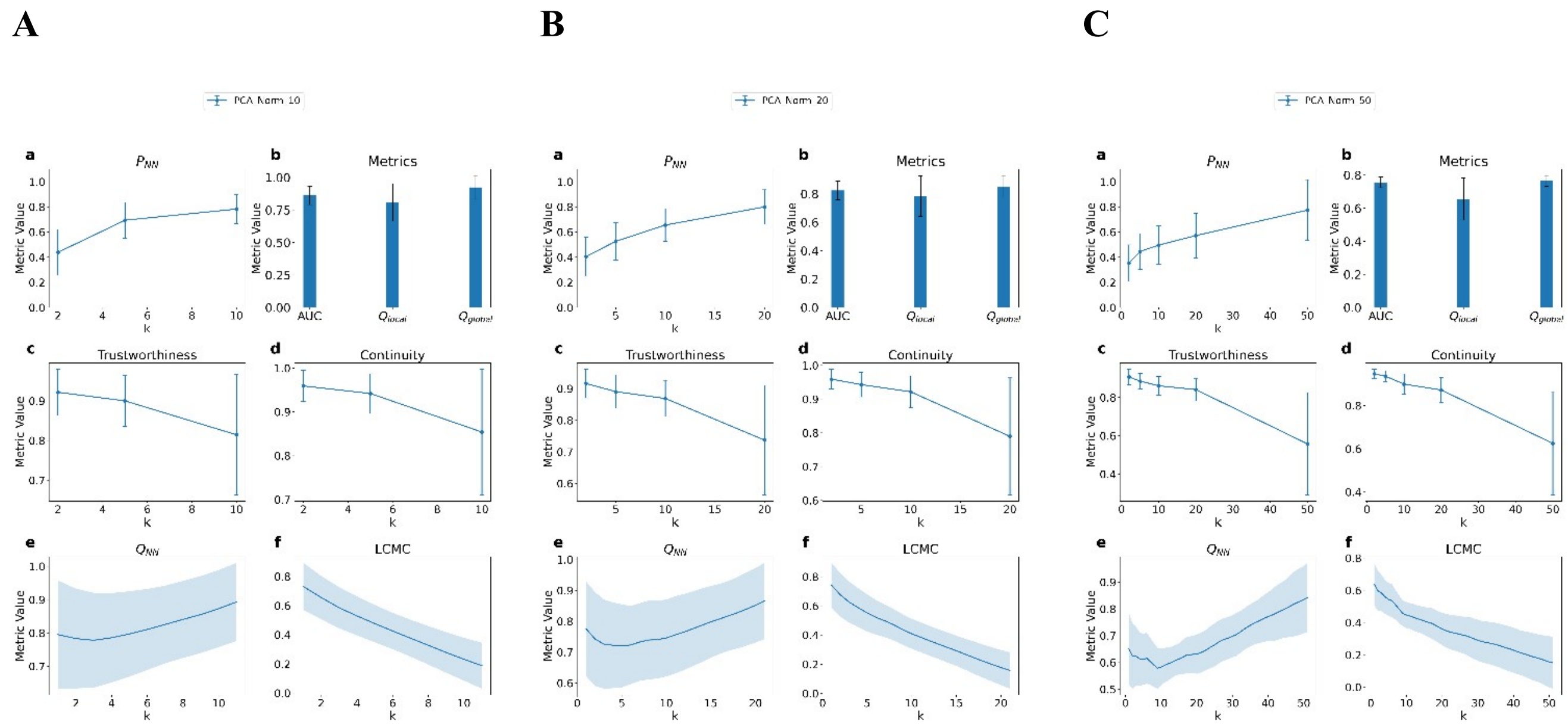

Figure S8: Detailed analysis of neighborhood metrics for the BitBIRCH-then-PCA on Normalized ECFP4 fingerprints for clusters having at least A) 11, B) 21 and C) 51 molecules.

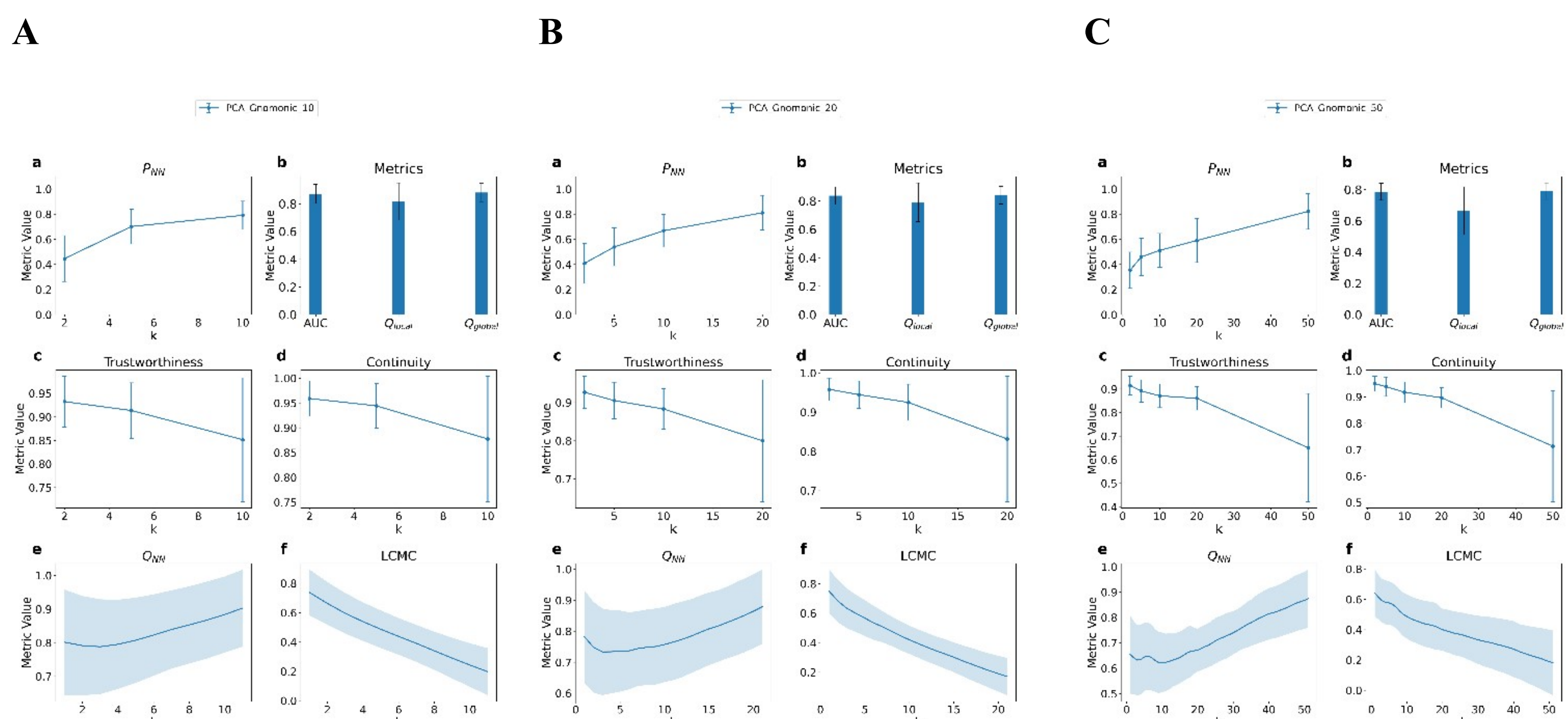

Figure S9: Detailed analysis of neighborhood metrics for the BitBIRCH-then-PCA on Gnomonic centroid projected fingerprints for clusters having at least A) 11, B) 21 and C) 51 molecules.

S5. A closer look at the distribution of metrics across clusters

The observed trend in neighborhood metrics across different molecular representations and projection methods affirm the robustness of BitBIRCH-PCA pipeline for chemical space visualization. This linear approach consistently yields lower information loss compared to non-linear alternatives like UMAP, providing a fast and reliable option, especially for large chemical datasets and scenarios where hyperparameter tuning poses practical challenges. To further refine these insights, a final granular analysis was performed by examining the distribution of neighborhood preservation metrics at the individual cluster level.

Figure S10 illustrates these distributions across all the clusters (ordered in decreasing order of cluster size). Out of all the metrics, AUC most clearly differentiates the two methods, with BitBIRCH-PCA exhibiting consistently superior performance for most clusters. On the other hand, local metrics like P_{NN}, Trustworthiness and Continuity reveal a more nuanced picture: for the largest clusters, both methods perform comparably, but BitBIRCH-PCA increasingly outperforms BitBIRCH-UMAP as cluster size decreases. Collectively, the evidence positions BitBIRCH-PCA as a robust, linear alternative matching UMAP's (best hyperparameters) projection quality for larger clusters while surpassing it for smaller ones, all while offering significant practical advantages-speed, reproducibility and lack of dataset-specific hyperparameter dependence, making it a suitable choice for large-scale chemical data visualization.

A

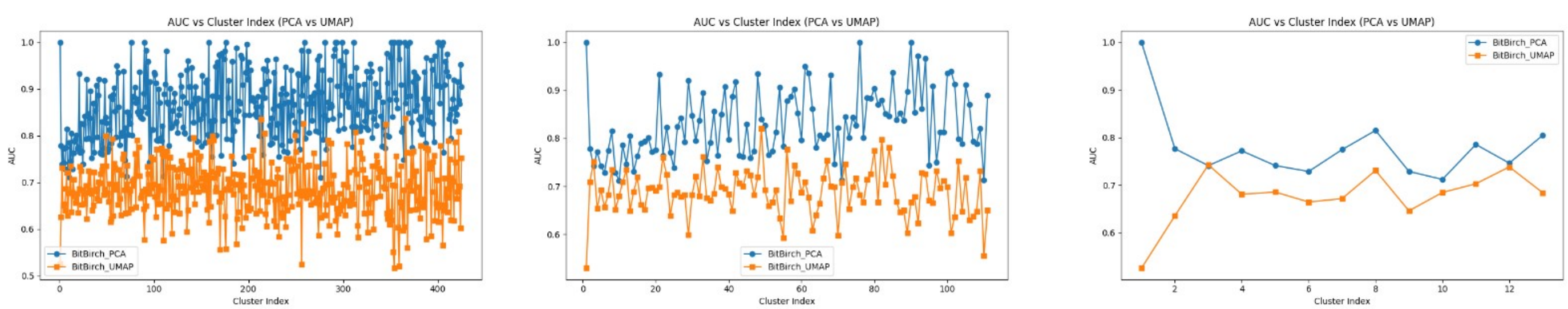

B

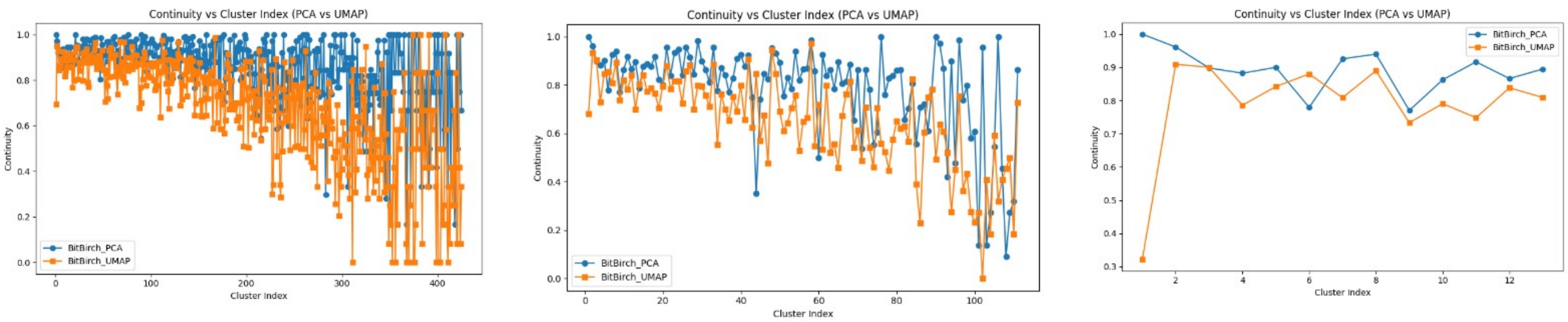

Continuity vs Cluster Index (PCA vs UMAP)
Continuity
Cluster Index
BitBirch_PCA
BitBirch_UMAP
Continuity vs Cluster Index (PCA vs UMAP)
Continuity
Cluster Index
BitBirch_PCA
BitBirch_UMAP
Continuity vs Cluster Index (PCA vs UMAP)
Continuity
Cluster Index
BitBirch_PCA
BitBirch_UMAP

C

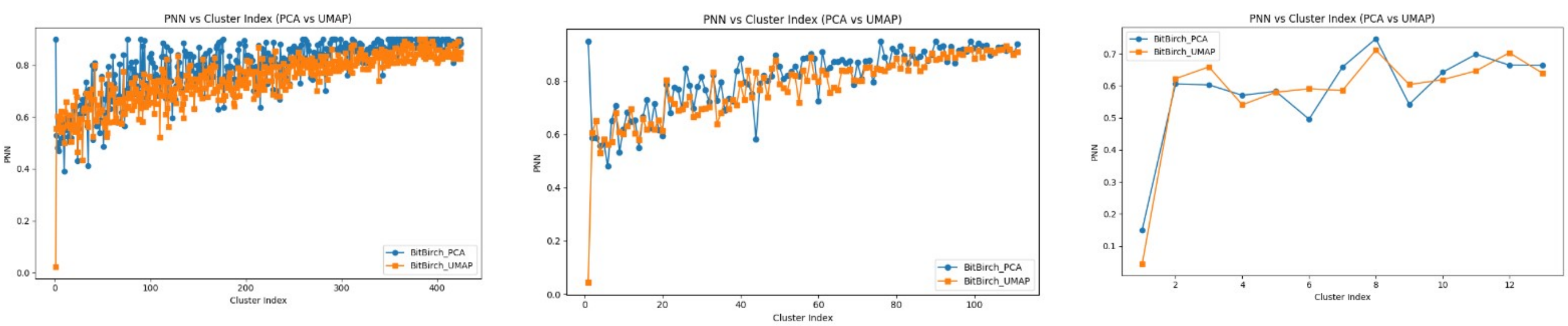

PNN vs Cluster Index (PCA vs UMAP)
PNN
Cluster Index
BitBirch_PCA
BitBirch_UMAP
PNN vs Cluster Index (PCA vs UMAP)
PNN
Cluster Index
BitBirch_PCA
BitBirch_UMAP
PNN vs Cluster Index (PCA vs UMAP)
PNN
Cluster Index
BitBirch_PCA
BitBirch_UMAP

D

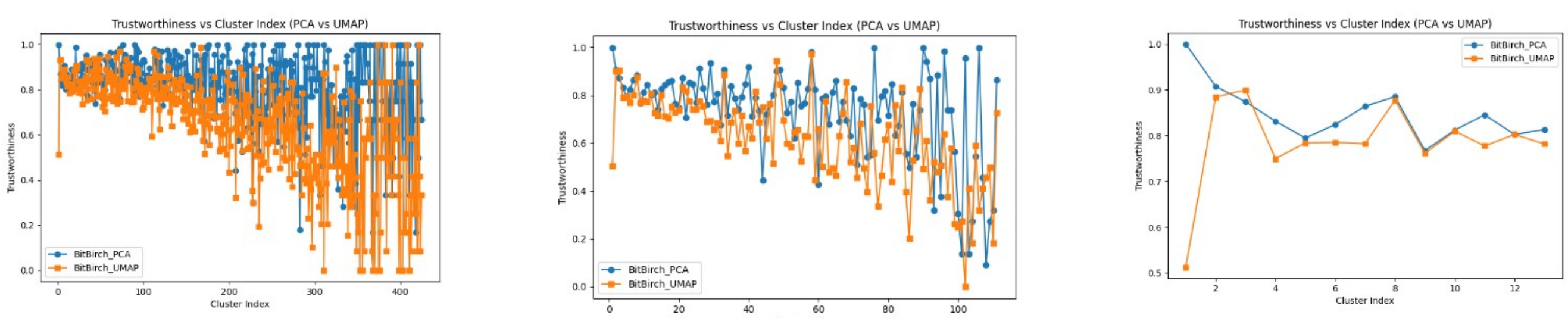

Trustworthiness vs Cluster Index (PCA vs UMAP)
Trustworthiness
Cluster Index
BitBirch_PCA
BitBirch_UMAP
Trustworthiness vs Cluster Index (PCA vs UMAP)
Trustworthiness
Cluster Index
BitBirch_PCA
BitBirch_UMAP
Trustworthiness vs Cluster Index (PCA vs UMAP)
Trustworthiness
Cluster Index
BitBirch_PCA
BitBirch_UMAP

Figure S10: Variation of DR metrics: A) AUC, B) Continuity, C) PNN and D) Trustworthiness across individual clusters for BitBIRCH-PCA (blue) and BitBIRCH-UMAP (orange). Clusters indices are arranged in decreasing order of cluster population.

S6. Clustering metrics at high similarity thresholds

A

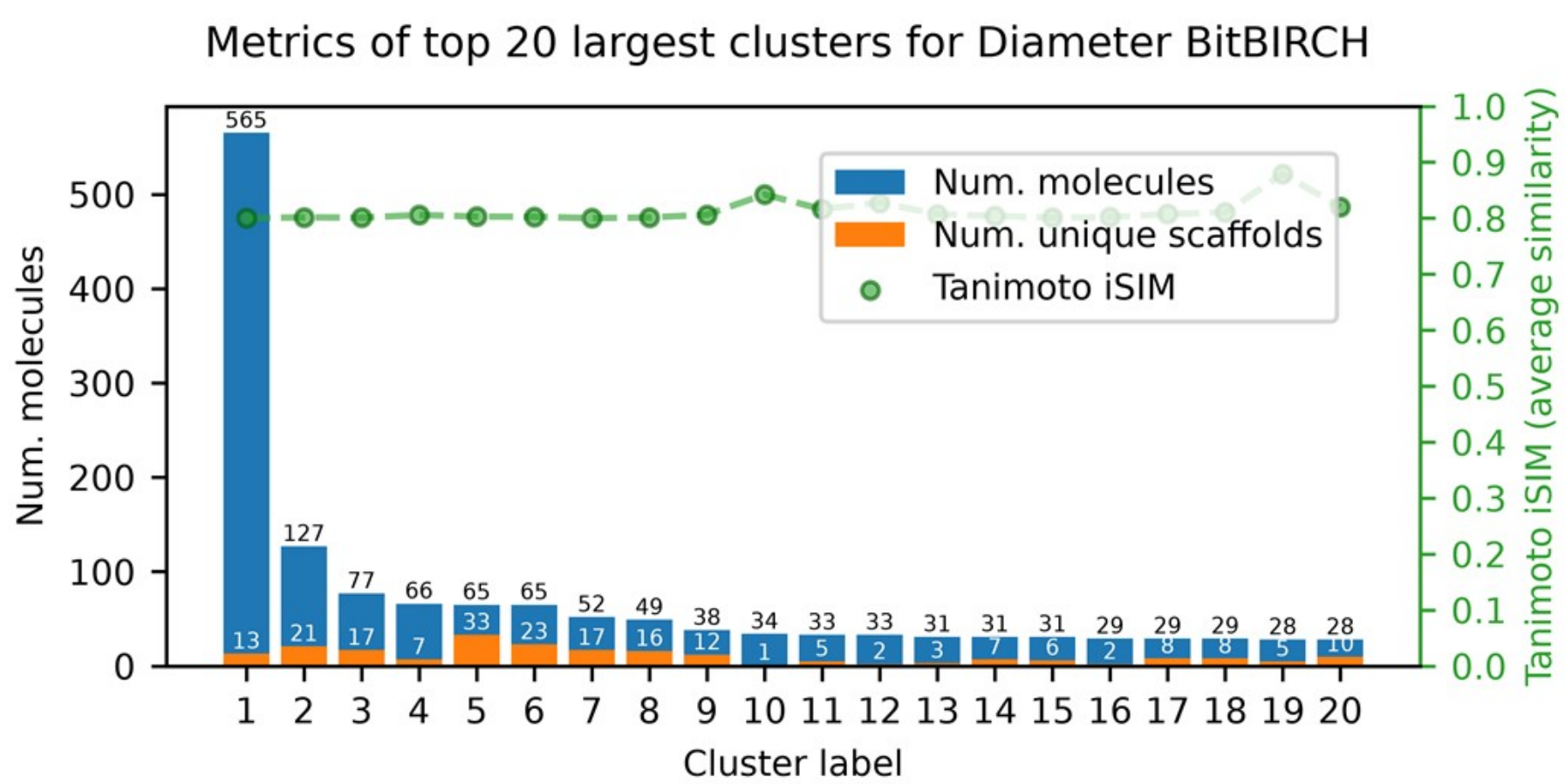

B

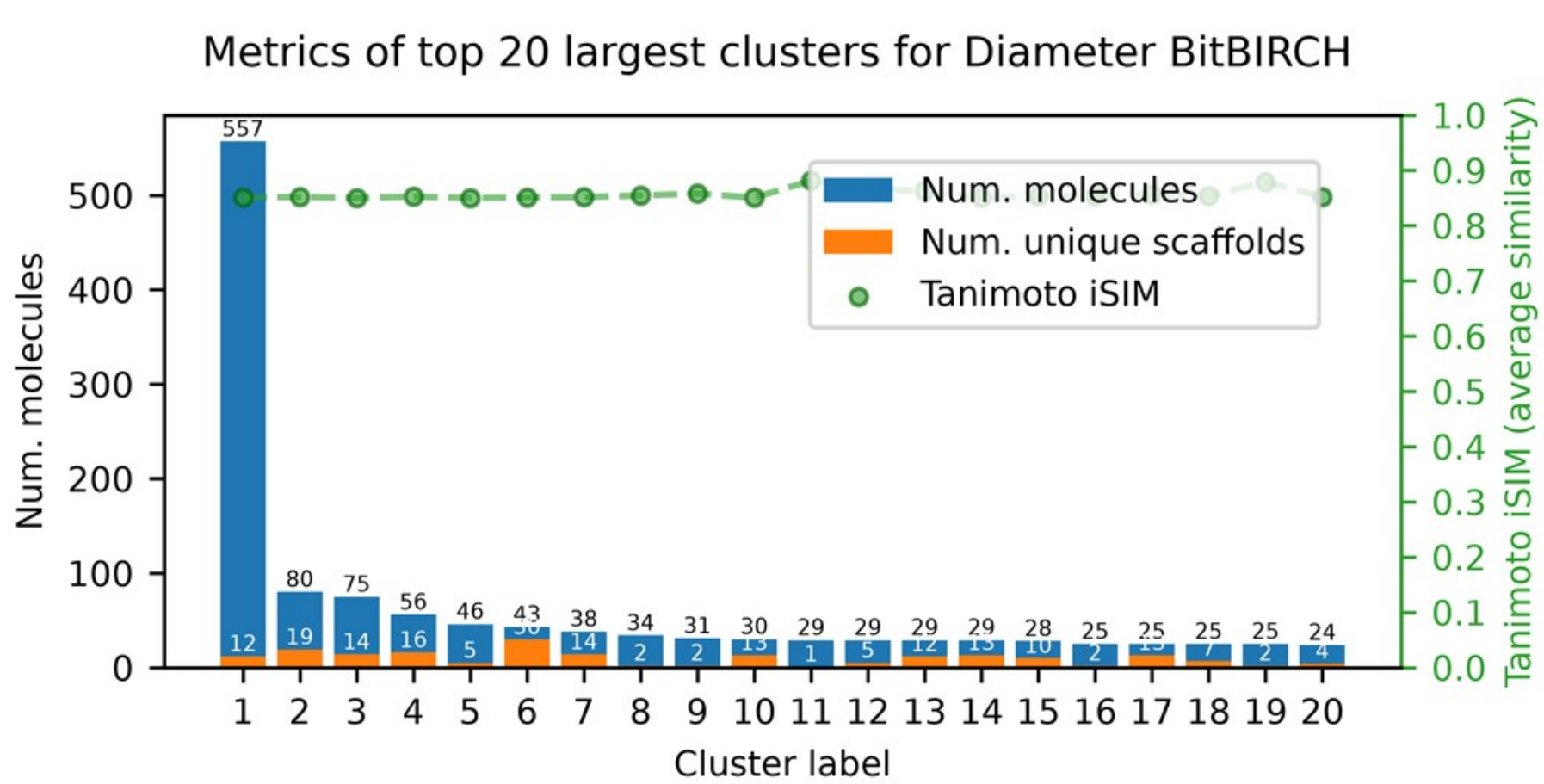

C

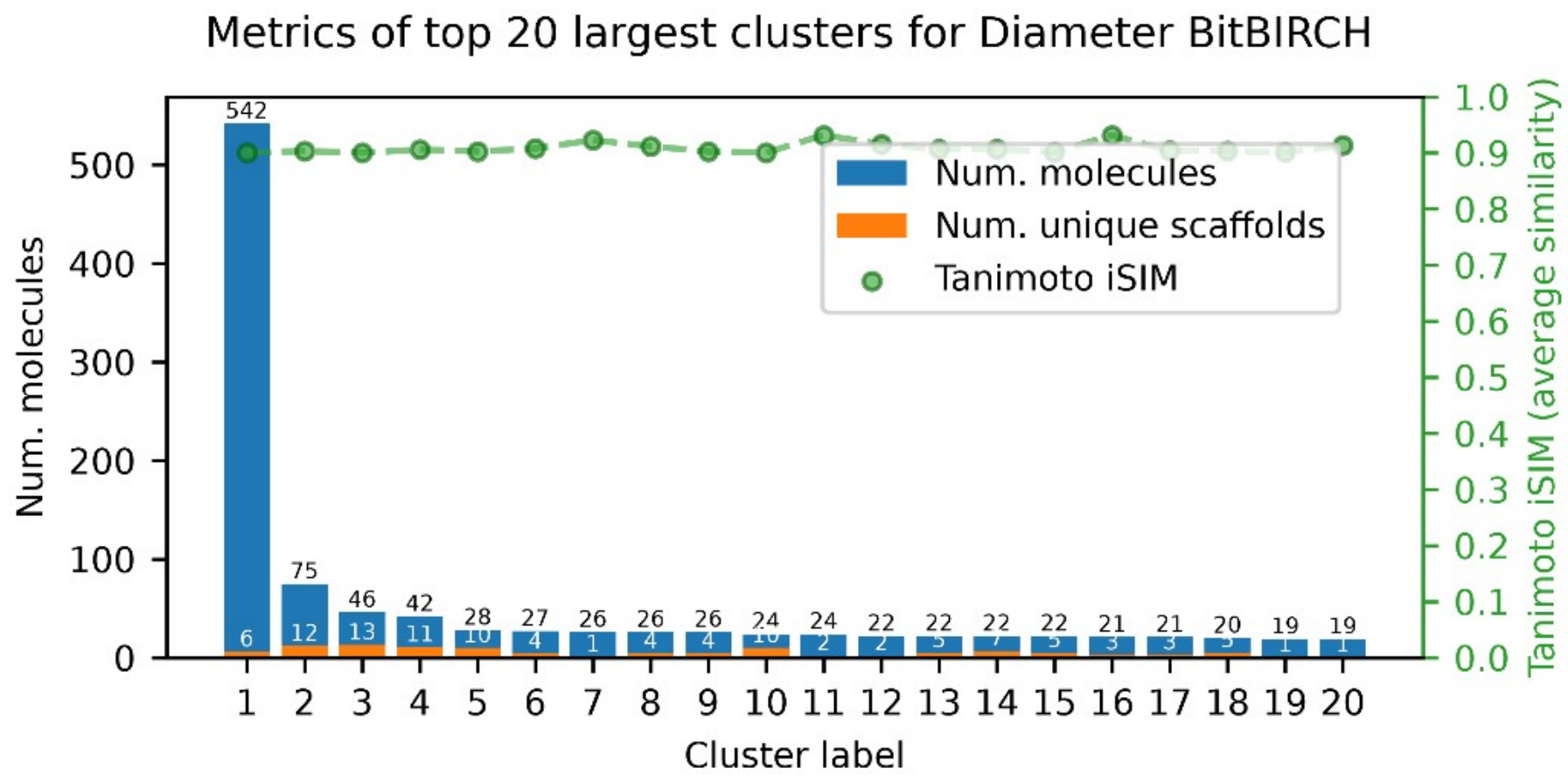

Figure S11: Bemis-Murcko scaffold and intra-cluster iSIM for BitBIRCH clusters at similarity thresholds **A.** 0.8, **B.** 0.85, **C.** 0.9
